# Comparative genomics of *Geobacter *chemotaxis genes reveals diverse signaling function

**DOI:** 10.1186/1471-2164-9-471

**Published:** 2008-10-09

**Authors:** Hoa T Tran, Julia Krushkal, Frances M Antommattei, Derek R Lovley, Robert M Weis

**Affiliations:** 1 Department of Chemistry, University of Massachusetts, Amherst, MA 01003, USA; 2 Department of Preventive Medicine, University of Tennessee Health Science Center, Memphis, TN 38163, USA; 3 Sharon Woods Technical Center, Procter and Gamble, Cincinnati, OH 45040, USA; 4 Department of Microbiology, University of Massachusetts, Amherst, MA 01003, USA

## Abstract

**Background:**

*Geobacter *species are δ-*Proteobacteria *and are often the predominant species in a variety of sedimentary environments where Fe(III) reduction is important. Their ability to remediate contaminated environments and produce electricity makes them attractive for further study. Cell motility, biofilm formation, and type IV pili all appear important for the growth of *Geobacter *in changing environments and for electricity production. Recent studies in other bacteria have demonstrated that signaling pathways homologous to the paradigm established for *Escherichia coli *chemotaxis can regulate type IV pili-dependent motility, the synthesis of flagella and type IV pili, the production of extracellular matrix material, and biofilm formation. The classification of these pathways by comparative genomics improves the ability to understand how *Geobacter *thrives in natural environments and better their use in microbial fuel cells.

**Results:**

The genomes of *G. sulfurreducens, G. metallireducens*, and *G. uraniireducens *contain multiple (~70) homologs of chemotaxis genes arranged in several major clusters (six, seven, and seven, respectively). Unlike the single gene cluster of *E. coli*, the *Geobacter *clusters are not all located near the flagellar genes. The probable functions of some *Geobacter *clusters are assignable by homology to known pathways; others appear to be unique to the *Geobacter *sp. and contain genes of unknown function. We identified large numbers of methyl-accepting chemotaxis protein (MCP) homologs that have diverse sensing domain architectures and generate a potential for sensing a great variety of environmental signals. We discuss mechanisms for class-specific segregation of the MCPs in the cell membrane, which serve to maintain pathway specificity and diminish crosstalk. Finally, the regulation of gene expression in *Geobacter *differs from *E. coli*. The sequences of predicted promoter elements suggest that the alternative sigma factors σ^28 ^and σ^54 ^play a role in regulating the *Geobacter *chemotaxis gene expression.

**Conclusion:**

The numerous chemoreceptors and chemotaxis-like gene clusters of *Geobacter *appear to be responsible for a diverse set of signaling functions in addition to chemotaxis, including gene regulation and biofilm formation, through functionally and spatially distinct signaling pathways.

## Background

Chemotaxis is a trait shared by many bacteria that enables cells to move toward chemical attractants and away from repellents. The chemotaxis system of *E. coli *regulates flagellar-based motility; it has been studied in the great detail and has served as a paradigm for chemotactic motility [1,2]. However, it is now apparent from genomic, genetic and biochemical studies conducted with other bacteria that a diversity of pathway functions and purposes exist well beyond the *E. coli *paradigm [3-5].

The *E. coli *chemotaxis pathway includes 11 genes, most of which are organized in a cluster near the flagellar genes [6]. This cluster contains two of the five genes for the transmembrane chemoreceptors, which are also known as methyl-accepting chemotaxis proteins (MCPs), and a single gene for each of the chemotaxis signaling proteins: the autophosphorylating histidine kinase (CheA), a scaffold protein (CheW), a methyltransferase (CheR), a methylesterase (CheB), a response regulator (CheY), and a CheY phosphatase (CheZ). The other three MCP genes are distantly located in the genome. Chemotactic signals are detected by a membrane array of MCPs, to which CheW and CheA are bound, and regulate CheA-mediated phosphorylation of CheY and CheB. By binding to the flagellar motor protein, FliM, CheY phosphate (CheY~P) induces swimming *E. coli *to tumble, which has the effect of reorienting the direction of swimming. CheB~P reduces kinase activity by demethylating the MCPs, which reduces the rate of CheY~P (and CheB~P) formation, and consequently reduces the cell tumbling frequency. The tumble-promoting activity of CheY~P is also extinguished by the action of CheZ. Overall, this stimulus-response pathway biases swimming motion of *E. coli *toward attractants and away from repellents. Adaptation to stimuli, mediated by the reversible methylation of MCPs in the process catalyzed by CheR and CheB, allows cells to remain sensitive to small changes in chemoeffector concentration over a large range [7,8].

Analyses of bacterial genome sequences show that homologs of chemotaxis genes are widespread [3,7]. From these surveys, it is apparent that the MCP and *che *genes in *E. coli *are relatively few in number, which may plausibly reflect modest requirements for sensory transduction in the environment that *E. coli *inhabits. By comparison, the chemotaxis-like systems in other bacteria are greater in number and diversity [4,5]. The copies of the 'core' genes, *e.g. cheAWY*, are clustered in multiple distinct locations and additional genes are present (*cheC, cheD, cheV *and *cheX*) that generate greater mechanistic diversity [5]. For example, Armitage and colleagues have shown that two chemotaxis clusters in the genome of *Rhodobacter sphaeroides *play a role in chemotaxis [9], an observation that plausibly reflects the greater need for different signaling pathways in complex environments. Pertinent to the analysis that we present below, is the fact that *Geobacter *sp. also occupies complex ecological niches in sedimentary environments. The published genome of *Geobacter sulfurreducens *has 34 MCP genes and six major *che *gene clusters [10]; these pathways are likely to play an important role in environmental adaptation.

Biochemical, genetic and physiological investigations of chemotaxis-like signaling pathways in bacteria other than *E. coli *have led to the realization that some of these pathways carry out functions distinct from the well-established role in regulating flagellar motor rotation. These functions include regulation of type IV pili-dependent motility, the expression of the motility apparatus (both flagella and type IV pili) and biofilm formation. As examples, *Pseudomonas aeruginosa*, *Rhodospirillum centenum*, *Myxoccocus xanthus*, and *Synechocystis *sp. all have multiple chemotaxis-like operons that have provided new insight into their diverse functions. *P. aeruginosa *has four major *che *clusters; two are involved in chemotaxis with different suggested roles, a third that regulates type IV pili motility and biosynthesis, and the fourth is involved in biofilm formation [11-16]. *R. centenum *has three *che *clusters; one mediates chemotaxis, a second regulates cyst development, and a third regulates flagellar synthesis [17-19]. *M. xanthus *has eight clusters; the functions for four clusters have been identified to date [20]. Each cluster regulates a different function, including cell motility, biosynthesis of the motility apparatus, or regulation of developmental genes [21-25]. The functions for two of the three clusters found in the genome of *Synechocystis *PCC6803 have been identified: one regulates type IV-dependent motility, the other pilus biosynthesis [26]. In a final example, only one of three *che *clusters in the *Vibrio cholera *genome regulates chemotaxis. Mutations in the two remaining clusters do not affect chemotaxis; their functions are yet to be identified [27].

*Geobacter *species are Gram-negative δ-*Proteobacteria *and are predominant in the Fe(III)-reduction zone of sedimentary environments. The ability to remediate subsurface environments contaminated with organics or metals and to produce electricity from waste organic matter makes *Geobacter *attractive species for further study [28,29]. *Geobacter *species are facultative anaerobes that can oxidize organic compounds completely to CO_2 _by using metal ions, *e.g*. Fe(III), Mn(IV) and U(VI), or electrodes as electron acceptors [30]. Most of the electron acceptors for *Geobacter *species are insoluble under environmental conditions. To overcome this constraint, they have developed mechanisms of electron transfer from the cell interior to the electron acceptors outside the cell. *Shewanella *and *Geothrix *use either chelators that solubilize metal oxides, or electron-shuttling compounds that transfer electrons from the cell surface to the insoluble acceptors [31,32]. *Geobacter *species use a different mechanism of electron transfer, in which the cells make direct contact with insoluble electron acceptors [33]. Cell motility and other processes that involve the synthesis of extracellular structures to mediate electron transfer (type IV pili and extracellular matrix materials) are critical for *Geobacter *species survival in the environment, and could potentially be regulated by chemotaxis-like pathways.

Clearly, flagella and pili play important roles in *Geobacter *physiology. When grown with insoluble metal oxides, *G. metallireducens *produce flagella and pili [34]. Moreover, it is postulated that flagellar-based motility and chemotaxis bring *G. metallireducens *cells to the metal oxide surfaces more efficiently, and that pili promote attachment and/or transfer electron [34]. In *G. sulfurreducens*, it has been demonstrated that the cells make direct contact with insoluble oxides *via *nanowires, pili that are electrically conductive and are essential for oxide reduction [33], and recent evidence suggests that this mechanism is more widespread than first thought [35]. Other than a direct role in electron conduction, pili, and the other extracellular matrix molecules that are involved in biofilm formation, are being studied for the role they play in efficient electricity production in microbial fuel cells [36,37].

The diverse functions of chemosensory systems in other bacteria suggest intriguing roles for the *Geobacter *chemotaxis and chemotaxis-like pathways. Therefore, we conducted an analysis of the chemotaxis gene homologs in three *Geobacter *species with completed genome sequences: *G. sulfurreducens*, *G. metallireducens *and *G. uraniireducens *as an initial step to understand their cellular functions better. All three genomes were found to contain an abundance of *che *gene homologs, which were organized into six to seven gene clusters and subdivided into predicted operons. The chemoreceptor (MCP) genes have a different organization. While several are located in *che *clusters, most are dispersed throughout the genome – this organization is typical of genomes that contain numerous MCP genes, *e.g. M. xanthus *[20]. We predict the functions of a number of the *Geobacter *gene clusters by comparisons to clusters of known function in other species. We have also found clusters, which are, to our knowledge, unique to the *Geobacter *species; these may be particularly important to *Geobacter *physiology.

## Methods

For protein sequence similarity searches, NCBI protein BLAST and position-specific-iterated-BLAST (blastp and psi-blast, respectively, ) [38] were used with default parameter values against the genomes of *G. sulfurreducens *PCA, *G. metallireducens *GS-15, and *G. uraniireducens *Rf4 (GenBank accession numbers AE017180.1, CP000148.1 and CP000698.1, respectively, ). To identify the *Geobacter *homologs of chemotaxis genes, *E. coli, B. subtilis *and *Thermotoga maritima *chemotaxis proteins were used as the test sequences, because these proteins are well-studied representatives, and are listed in the curated databases [39-41]. The following sequences were used: the *E. coli *aspartate receptor methyl-accepting (MA) domain (residues 267–514) (gi|16129838), the complete sequences of *E. coli *CheA (gi|1788197), CheB (gi|16129835), CheR (gi|16129836), CheW (gi|16129839), CheY (gi|16129834) and CheZ (gi|16129833); the complete sequences of *B. subtilis *CheC (gi|2634017), CheD (gi|2634018) and CheV (gi|2633772), and the complete sequence of *T. maritima *CheX (gi|81553634). ClustalW  was used with default values for the parameters to conduct multiple sequence alignments to determine percent identities and to establish the class membership of the methyl-accepting domains [42]. TMHMM2 [43], TmPred [44], and TopPred [45] were used (with parameters set to default values) to predict the number of transmembrane helices in the putative methyl-accepting chemotaxis proteins. A polypeptide segment was designated a transmembrane α-helix when at least two of the three programs identified the same polypeptide segment as a transmembrane helix. Phylip (version 3.6) was used to construct CheA and CheY phylogenetic trees by the neighbor-joining method [46,47], as implemented in NEIGHBOR. SEQBOOT was used to generate 1000 bootstrap replicates and pairwise distances were estimated with PRODIST. The JTT model was used with no among-site variation. The trees were left unrooted.

The organization of *che *gene operons in the *Geobacter *sp. was predicted with FGENESB (Softberry Inc., ). FGENESB identifies protein-coding genes with Markov chain models of coding regions and translation start and termination sites, and annotates them with information from public databases. The sequence parameters (coding content, oligonucleotide composition, and gene length distribution) were estimated in FGENESB for each genome separately through an iterative procedure with the minimum ORF length set to 100 nt. Additional features, *e.g*. tRNA and rRNA, σ^70 ^family promoters, and rho-independent terminators, were predicted from sequence similarity, linear discriminant analysis, or modeling approaches. FGENESB-based operon predictions were generated from the directions of adjacent genes, the distribution of intergenic distances, the presence or absence of predicted promoter and terminator regions, and the conservation of pairs of adjacent genes across microbial genomes (V. Solovyev, *personal communication*). The operon annotation of the *G. sulfurreducens *genome used in this study has been described previously [48], and is available online [49].

σ^54^-regulated promoters were predicted from a search of the *G. sulfurreducens *genome with PromScan [50]. This software assigns a score representing the Kullback-Leibler distance, based on 186 known sites from 47 bacterial species [51]. The *G. sulfurreducens *genome was found to contain 110 predicted σ^54^-regulated promoters with a score equal to or greater than 80 (the default value) in noncoding regions upstream of genes and operons. The current accuracy of prediction is 78%, an estimate obtained from experiments that positively identified 14 RpoN-dependent regulation out of 18 predicted sites (J. Krushkal, C. Leang, M. Puljic, T. Ueki, R. Adkins, and D. Lovley, *unpublished results*). In addition, PromScan was used to look for σ^54^-regulated promoters upstream of the major *che *clusters in the *G. metallireducens *and *G. uraniireducens *genomes. Finally, putative σ^28^-regulated promoters upstream of the flagellar filament gene (*fliC*) and the major *che *clusters in the genomes of *G. sulfurreducens*, *G. metallireducens *and *G. uraniireducens *were identified with Virtual Footprint [52] and the Neural Network Promoter Prediction software for bacterial species [53]. Five hundred base pairs upstream of the putative initiation codons of genes of interest were analyzed using default parameters.

## Results and discussion

### *Geobacter* Chemotaxis Genes: Numbers and Organization

**Figure 1:**
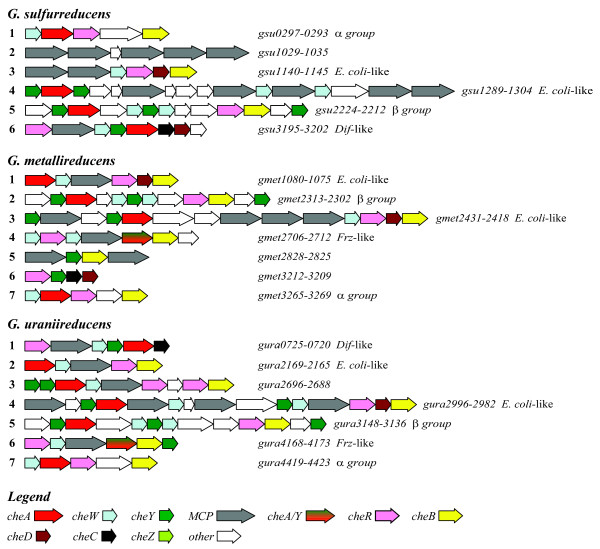
**Physical arrangement of the major *Geobacter *chemotaxis-like gene clusters**. Affiliations with *che *clusters of known function are indicated after the clusters, as *E. coli*-like, *Dif*-like and *Frz*-like (both from *M. xanthus*), and the α and β *group*s. These assignments were made by the relative agreements between *che *gene content, the physical arrangement in the cluster and the percent identities. The α and β *group *designations refer to *che *clusters that are unique to the *Geobacteraceae *and the δ-*Proteobacteria*, respectively.

BLAST analysis of the *G*. *sulfurreducens*, *G*. *metallireducens*, and *G*. *uraniireducens *genomes identified multiple copies of the chemotaxis genes; over 60 genes in each species were homologous to the known *che *and *mcp *genes in *E. coli*, *B. subtilis *and *T. maritima *(Table 1). Homologs of all the *che *genes from *E. coli *were present in the *Geobacter *species, except *cheZ*, which is found much more frequently in genomes of β- and γ-proteobacteria in comparison to the genomes of α-, ε-, and especially δ-proteobacteria [54]. The *Geobacter *genomes also contained *cheC*, *cheD*, *cheV*, and *cheX *homologs. With the exception of the genes for the chemoreceptors – the methyl-accepting chemotaxis proteins (MCPs), which were dispersed throughout the genomes, most of the *che *genes were clustered, as shown in Figure 1. In some cases, additional genes encoding hypothetical proteins of unknown function or annotated proteins with functions not known to be involved in chemotaxis-related signaling pathways were located in these clusters. There are six major chemotaxis-related gene clusters in *G. sulfurreducens*, and seven major clusters each in *G. metallireducens *and *G. uraniireducens*; their physical arrangements are depicted in Figure 1. None of these clusters is located close to the flagellar gene clusters.

**Table 1:** Numbers of *che *gene homologs in *E. coli*, *B. Subtilis *and *Geobacter sp*.^*a*^

	**Species**				
	
**Gene**	***E. coli***	***B. subtilis***	***G. met*.**	***G. sul*.**	***G. ura*.**
*cheA*^*b*^	1	1	5	4	7
*cheB*	1	1	8	4	5
*cheR*	1	1	9	5	10
*cheW*	1	1	8	10	10
*cheY*^*c*^	1 (1)	1 (3)	10 (21)	7 (25)	10 (25)
*cheZ*	1	0	0	0	0
*cheC*	0	1	2	1	1
*cheD*	0	1	3	3	2
*cheX*	0	0	1	1	1
*cheV*	0	1	1	1	1
*mcp*	5	10	18	34	24
**Total**	**11**	**17**	**65**	**70**	**71**
No. of *che *clusters^*d*^	1	1	7	6	7

^*a*^The numbers of homologs were determined by blastp searches (with default values for the parameters).

^*b*^The number of *cheA *homologs in the genomes of *G. met*. and *G. ura*. includes a contribution from one *cheAY *fusion.

^*c*^The numbers are *cheY *genes in the major clusters. Numbers in parentheses also includes genes that encode for singleton CheY-like receiver domain proteins.

^*d*^Chemotaxis gene clusters are defined to contain three or more *che *genes.

**Figure 2:**
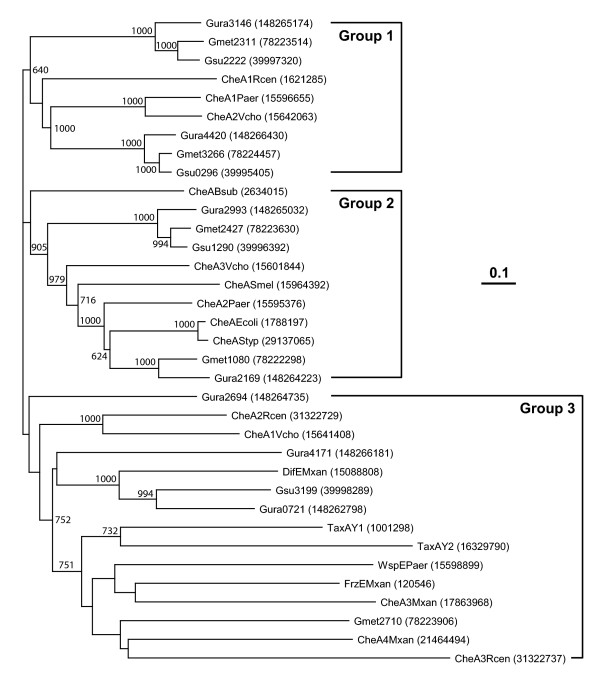
**Neighbor-joining tree of putative CheA homologs of *Geobacters *and CheAs from other well studied species**. These include *Escherichia coli *(Ecoli), *Bacillus subtilis *(Bsub), *Pseudomonas aeruginosa *(Paer), *Sinorhizobium meliloti *(Smel), *Rhodospirillum centenum *(Rcen), *Vibrio cholerae *(Vcho), *Myxococcus xanthus *(Mxan), *Salmonella typhimurium *(Styp) and *Synechocystis *sp. strain PCC6803 (Syne). The GenInfo Identifier protein sequence numbers are displayed in parentheses at right. All positions with gaps in the aligned sequences were excluded. Bootstrap values from 1000 replicates of >600 are shown in respective nodes. The tree figure was generated with TreeView, version 1.6.6 [96].

The genomes of *G. sulfurreducens*, *G. metallireducens *and *G. uraniireducens *code four, five and seven predicted *cheA *genes, respectively. The homologs encoded by the *cheA *genes are clustered in three groups of the phylogenetic tree (Figure 2), demonstrating that the multiple *cheA *genes did not result from recent gene duplication events, but are paralogs that have been evolving separately for some time, which suggests that they play distinct cellular roles. Each CheA homolog, together with the other cognate *che *gene products, is likely to regulate a separate chemotaxis-like pathway. The presence of multiple *che *homologs and clusters are a strong indication of different pathways that raise intriguing questions about function, and whether or not the pathways are redundant or exhibit crosstalk. By comparing the gene order and the percent identities of the gene products from other bacteria, in which chemotaxis and chemotaxis-like pathways are studied extensively, we were able to predict the functions for many of the *Geobacter che *clusters. From this analysis, it seems unlikely that different clusters constitute redundant pathways; instead, each pathway has a distinct function. In addition, plausible mechanisms to reduce unwanted crosstalk between pathways emerged.

The *Geobacter *genomes are predicted to have large numbers of standalone response regulators proteins that are comprised only of the receiver domain [55]; we refer to these as CheY-like proteins. The *G. metallireducens*, *G. sulfurreducens*, and *G. uraniireducens *genomes have 21, 25, and 25 homologs, respectively, but the majority is probably *not *involved in chemotaxis-like signaling [54]. Only 38% of the homologs are located in the major *che *or flagellar gene clusters (Table 1), the remainder (11, 18 and 15, respectively) are located elsewhere on the chromosome. Of those we suspect to play a role in chemotaxis-like signaling, *i.e*. the *cheY *genes that are located in the major *che *or flagellar gene clusters, about 50% reside in a branch of the phylogenetic tree with *E. coli *and *Salmonella *CheY (four apiece from *G. metallireducens *and *G. sulfurreducens*; five from *G. uraniireducens*, Figure S1) [see Additional file 1]. These CheY homologs are most likely to have response regulator functions as the substrates of CheA-mediated phosphorylation in chemotaxis pathways. The *Geobacter *CheY-homologs located elsewhere in the tree (relatively distant to *E. coli *and Salmonella *CheY*), but are situated in *che *or flagellar gene clusters on the chromosome, probably also function in chemotaxis-like pathways, perhaps in some other manner. By contrast, the genes encoding the most distantly-related CheY-like proteins, *i.e*. located outside *che *clusters, away from the flagellar genes, *and *are (relatively) distant to *E. coli *and *Salmonella *CheY in the phylogenetic tree (Figure S1) [see Additional file 1], probably function in other two-component pathways. For instance, *B. subtilis *and *Nostoc *CheY-like homologs, which are not in the *che *clusters, are involved in two-component pathways unrelated to chemotaxis [56,57]. Therefore, we postulate that the standalone receiver proteins encoded within the *Geobacter che *and flagellar gene clusters plausibly represent predicted CheYs with chemotaxis-like pathway function.

### Number and Diversity of *Geobacter* MCPs

The three *Geobacter *genomes investigated in this study were found to have significant numbers of genes for MCPs: 34 in *G*. *sulfurreducens*, 18 in *G*. *metallireducens*, and 24 in *G*. *uraniireducens *(Table 1). These putative MCPs were identified through the presence of the highly conserved methyl-accepting (MA) domain, which was first assigned a biochemical function in the *E. coli *chemoreceptors as the domain methylated in a CheR-dependent process [58,59]. The large number of MCP-coding genes in the *Geobacter *genomes, by comparison to either *E. coli *or *B. subtilis*, plausibly reflects a greater need to detect sensory stimuli in the subsurface environment. With the exception of the aerotaxis receptor, all *E. coli *MCPs have periplasmic ligand-binding domains that detect the external chemoeffector concentrations, two transmembrane (TM) helices, and the (juxtamembrane) HAMP and the methyl-accepting (MA) domains located in the cytoplasm. The sequences of the predicted *Geobacter *MCPs reveal significantly greater diversity in the domain organization and architecture of the sensing domains (Figure S2) [see Additional file 1].

The N-terminal regions of MCPs sense various environmental stimuli through diverse means, because the length and heterogeneity of these regions are greater, compared to the cytoplasmic domains, which are mostly organized like the *E. coli *MCPs (a single HAMP domain followed by the MA domain). Domain architectures of representative *Geobacter *MCPs are shown in Figure S2 and Table S1 [see Additional file 1]. With respect to transmembrane (TM) segments, the *Geobacter *MCPs fall into three groups according to the number of predicted TM helices (zero, one or two). Of the 76 predicted MCPs found in the genomes of *G. metallireducens*, *G. sulfurreducens *and *G. uraniireducens*, ninety percent have two TM helices. Most of these, 80%, have periplasmic domains that are ~150–200 amino acid residues (aa) in length, which are most similar in size to the periplasmic domains of the major *E. coli *MCPs. Three percent of the *Geobacter *MCPs have larger periplasmic domains (~250–430 aa), while the others have a significantly smaller domain (<100 aa). MCPs with the Tar-like and larger periplasmic domains probably detect signals through these domains by ligand binding. While the structures of most of these MCPs are not known, the sensory domains of two MCPs (*Gsu0935 *and *Gsu0582*) are PAS domains with covalently-bound hemes [60]. Interestingly, this places redox-active sensing domains in the periplasm. By contrast, the MCPs with small periplasmic domains are more likely to detect signals via associations with other proteins, as in the case of DifA of *M. xanthus *[24,61], or detect intracellular signals when the MCPs have no TM segments [4].

MCP MA domains were recognized to belong to a superfamily based on a multiple sequence alignment first conducted by Le Moual and Koshland [59]. A more recent analysis of approximately 2000 MCPs identified seven classes (named 24 H, 28 H, 34 H, 36 H, 38 H, 40 H and 44 H), which are defined by the number of heptad repeats (H) in the cytoplasmic domain [62]. The most well characterized MCPs of *E. coli*, Tar and Tsr, belong to class 36 H, and the MCPs from *T. maritima *(TM1143) and *B. subtilis *(McpA and McpC) belong to class 44 H. Multiple-sequence-alignments of the *Geobacter *MCPs revealed that *G. metallireducens *has MCPs in classes 24 H, 34 H, 36 H and 40 H; *G. sulfurreducens *has MCPs in classes 24 H, 34 H, 40 H and 44 H, and *G. uraniireducens *has MCPs in classes 24 H, 34 H, 36 H, 40 H and 44 H. The majority of the MCPs are members of class 34 H (17, 24, and 21% in *G. metallireducens*, *G. sulfurreducens *and *G. uraniireducens*, respectively) and class 40 H (61, 71 and 46%, respectively). [Additional file 2 is the multiple sequence alignment of the *Geobacter *MCPs.] *G. metallireducens *and *G. uraniireducens *each have one MCP in class 36 H (*Gmet1078*, *Gura2167*), and *G. sulfurreducens *and *G. uraniireducens *each have one MCP in class 44 H (*Gsu3196*, *Gura0724*).

MCPs and Che proteins form specific clusters. In *E. coli*, all the MCPs and most of the Che proteins are found in clusters that are often located at the cell poles [63-65]. When bacteria have two or more chemotaxis (or chemotaxis-like) gene clusters, the clusters are observed to have distinct locations and compositions [66-68]. We speculate that MCP class membership is a contributing factor of cluster specificity. According to this reasoning, MCPs in the same class are more likely to belong to the same cluster, and conversely, MCPs in different classes are likely to segregate. Cluster formation, in part, is generated by contacts between the MCP MA domains. The MA domain is a coiled-coil hairpin that dimerizes to form a long four helix bundle [69,70], where the bundle length is determined by the number of heptad repeats, ~210 Å for class 36 H MCP (*E. coli *Tsr) and ~260 Å for class 44 H MCP (*T. maritima *TM1143). We postulate that class-specific MCP clusters are more likely to form for the following reason: two different MCPs, which contain MA domains belonging to the same class, are more likely to engage in the interactions that lead to the formation of clusters than two MCPs that contain MA domains from different classes (and therefore different MA-domain lengths).

The localization of *P. aeruginosa *and *R. sphaeroides *protein clusters provide some support for class-specific cluster formation. *P. aeruginosa *McpB and WspA, which are found in distinct signaling clusters in distinct locations (polar and lateral, respectively), belong to different classes (36 H and 40 H, respectively) [66,67]. *R. sphaeroides *McpG and TlpT (a soluble MCP) belong to different MA classes (34 H and 36 H, respectively) and locate in different clusters (polar membrane and cytoplasmic locations, respectively) [68]. We anticipate that the multiple classes of MCPs present in the *Geobacter *species contribute to the formation of segregated MCP signaling clusters. On the other hand, MA class membership is certainly not the only factor to consider. For example, this mechanism cannot easily explain the localization of MCPs that do not belong to any class [62]. In addition, the compositions of signaling clusters are influenced undoubtedly by the specificity of interactions between the different MA domain and Che protein homologs. These effects (and others), considered together, can contribute to the assembly of specific signaling units, which function in the same cell without unwanted crosstalk.

### The Prevalence and Specificity of CheR Tethering Segments

The role of a semi-conserved pentapeptide at the C-terminus of some MCPs, and first observed in the *E. coli *high abundance receptors Tar and Tsr (NWETF) [71], has a well established role in sensory adaptation by mediating efficient receptor methylation and demethylation [7,71-74]. In the process of receptor methylation, the pentapeptide NWETF binds to the β-subdomain of CheR at a location that is distinct from the active site – methylation site interaction [75]; this interaction tethers CheR near the methylation sites of clustered receptors. It is plausible to expect that all MCPs containing the C-terminal NWETF or a pentapeptide similar to NWETF provide adaptational assistance via the mechanism established in *E. coli *[73,76]. MCPs that contain the CheR-binding pentapeptide are restricted primarily to the *Proteobacteria*; the genomes of bacteria in other phyla reveal few, if any, MCPs that contain a recognizable CheR-tethering segment, as defined previously [7]. In such species – for example *B. subtilis *and *T. maritima*, methylation operates through a different, pentapeptide-independent mechanism [77]. Less than 10% of the ~2500 MCPs listed in the SMART database of completed bacterial genomes contain a recognizable CheR tethering segment; this segment always follows the MA domain (SM00283) in the primary sequence of the MCP, which then ends in a pentapeptide that binds CheR [7]. Therefore, many MCPs are probably methylated and demethylated *via *a pentapeptide-independent mechanism.

Closer analysis of all the MCPs that contain the NWETF pentapeptide or a similar pentapeptide, reveal a restricted class membership, either to class 34 H or to class 36 H [62]. 85% of these MCPs belong to class 36 H *and *contained the class-specific xWxxF pentapeptide motif; 15% belong to class 34 H *and *contained the class-specific xF/YxxF/Y motif for the pentapeptide [7]. In contrast to the kingdom-wide percentages, most pentapeptide-containing *Geobacter *MCPs belonged to class 34 H (100%, 75%, and 80% for *G. sulfurreducens*, *G. metallireducens *and *G. uraniireducens*, respectively). *G. metallireducens *and *G. uraniireducens *have one *mcp *gene apiece in the class 36 H with a C-terminal DWKEF pentapeptide, a sequence more similar to the *E. coli *consensus (NWETF). Using the pentapeptide-containing MCPs as one criterion, we defined the *che *clusters to which these *mcp *genes belong as '*E. coli*-like' clusters (Figure 1).

**Figure 3:**
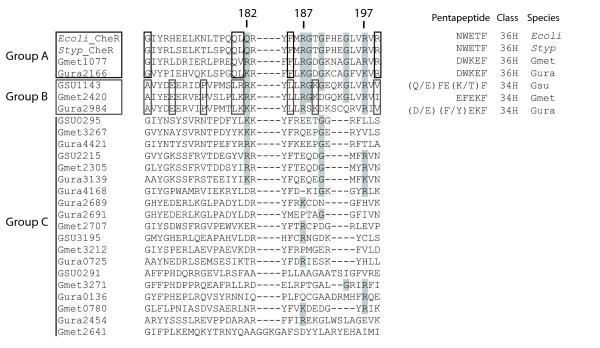
**Alignment of the beta-subdomain of *Geobacter *CheR homologs with *E. coli *and *S. enterica *CheR**. Based on homology, the *Geobacter *CheRs were divided into three groups. Two groups (A and B) displayed significant identity with residues important for binding pentapeptide (highlighted in grey) and the third group (C) did not. Gene positioning provides further evidence that the group A and B homologs bind to MCPs containing the C-terminal pentapeptide: these homologs are located in *che *clusters with pentapeptide-containing MCPs (Figure 1). Group A consists of two CheR homologs that are located near two class-36 H MCPs. The consensus pentapeptide of the MCPs that are cognate to the Group B CheR homologs, EFEKF, is found in class-34 H MCPs.

To identify possible class-specific MCP-methyltransferase tethering interactions, we compared the aligned β-subdomain sequences of the *Geobacter *CheR homologs to the *Salmonella *and *E. coli *CheR sequences. The *Salmonella *CheR structure, co-crystallized with the NWETF pentapeptide has enabled the identification of residues in the β-subdomain that are involved in the peptide-CheR interaction (Q182, G188, R187, G190, G194 and R197, numbered according to *Salmonella *CheR, PDB# 1bc5) [75,77]. Figure 3 shows aligned sequences from the β-subdomain of all the *Geobacter *CheR homologs, together with the *E. coli *and *Salmonella *sequences (residues 166–199). Using this alignment, we divided the *Geobacter *CheRs into three groups. Two groups (A and B) displayed significant identity with residues important for binding a pentapeptide; the third and largest group (C) did not (Figure 3). Consequently, we concluded that the CheR homologs in Group C probably do not methylate MCPs by the *E. coli *mechanism.

The colocalization of *mcp *and *CheR *genes within the same clusters provided evidence that group A and B CheR homologs bind to MCPs containing a C-terminal pentapeptide; these CheR homologs are located in *che *gene clusters containing at least one gene that encodes a pentapeptide-containing MCP (Figure 1). The two CheR homologs that comprise group A are located adjacent to class-36 H MCPs (*Gmet1078*, *Gura2167*) and have DWKEF as the C-terminal pentapeptide – judged to be more similar to the *E. coli *consensus (NWETF). By contrast, the consensus pentapeptide coded by *mcp *genes located in the *che *gene clusters with group B CheR homologs is EFEKF. All 14 MCPs that contain this consensus pentapeptide belong to class 34 H (Figure 3) [also see Additional file 2], and 10 of these are located in the *che *gene clusters that contain the group B *CheR *genes (Figure 1). Differences in the consensus pentapeptide for class 34 *versus *class 36 MCPs correlate with differences in the β-subdomain amino acid residues in pentapeptide-binding pocket of group A *versus *B CheR homologs (respectively, Figure 3). Thus, it is plausible that these differences contribute to (and reflect) class-specific MCP-CheR interactions.

By contrast, all group C CheR homologs are either (a) not located in a *che *gene cluster, (b) located in *che *clusters that do not contain an *mcp *gene, or (c) located in *che *clusters that contain genes encoding class 40 H or 44 H MCPs, These MCPs do not contain recognizable CheR tethering segments (terminating in a pentapeptide) according to criteria defined previously [7]. Thus, it is probable that group C CheRs use a pentapeptide-independent mechanism for receptor methylation, similar to that observed with *T. maritima *[77]. In addition, we interpret the specific pairings within the *che *gene clusters, of the CheR groups (A, B, C) and the MCP classes (36 H, 34 H, 40 H/44 H, respectively), as support for the idea of class-specific receptor signaling.

### Predicted Function of *E. coli*-like Chemotaxis Clusters

Above, we defined *Geobacter che *clusters operationally as '*E. coli*-like' by the presence of one or more *mcp *genes that encode MCPs with CheR tethering segments. In addition, the *Geobacter *CheA homologs in these clusters belong to the same phylogenetic grouping as *E. coli *CheA (Figure 2). These clusters were sorted further into two types. Type 1 clusters – clusters one and two in the *G. metallireducens *and *G. uraniireducens *genomes, respectively (Figure 1), have significant resemblances to the *E. coli mocha-meche *cluster, judged by the gene order and by the percent identities between predicted *Geobacter *proteins and the *E. coli *proteins (Figure 2 and Figure S3) [see Additional file 1]. Notably, the *Geobacter mcp *genes in these two clusters encode for MCPs that belong to class 36 H, the same as *E. coli *MCPs.

Type 2 clusters are also characterized by significant sequence identity (although lower than Type 1), but the gene positions bear a comparatively small resemblance to the *E. coli *cluster. Moreover, the Type 2 clusters contain predicted ORFs in significant numbers that have unknown function or assigned functions other than chemotaxis. (See Figure S3 [Additional file 1] for comparisons of gene arrangement and the percent identities of individual gene products.) The Type 2 clusters possess multiple genes coding for MCPs that belong to class 34 H; many of these contain a CheR-tethering segment that terminates in an 'NWETF-like' pentapeptide at the C-terminus.

Three features distinguish the *E. coli*-like *Geobacter che *clusters from the *E. coli *cluster. (*1*) The *E. coli meche *operon contains *cheZ*, but *Geobacter *genomes do not, so a CheZ-independent signaling mechanism must operate in *Geobacter *pathways. (*2*) Multiple CheW genes are found in each cluster (except for *G. metallireducens*), an observation made previously with other bacteria. Studies of the CheW homologs in *R. sphaeroides *have led to the suggestion that these homologs do not perform redundant functions, but engender MCP-specific interactions, a proposal based on observed differences in binding affinity [9]. It has also been suggested that multiple CheWs allow additional MCPs to be incorporated within the chemosensory system, since there tends to be more *mcp *genes than *cheW *genes [11]. Another interesting hypothesis has been proposed: the different CheWs may recognize MCPs in a class-specific manner, which produces different specific signaling pathways in a *che *cluster [54]. (*3*) Finally, *cheD *and other non-*che *genes – not found in the *E. coli *chemotaxis cluster, are present in the *E. coli*-like *Geobacter *Type 2 clusters. By analogy to the functions assigned in *B. subtilis *and *T. maritima*, the presence of CheD signifies that a different mechanism is in play for deamidating MCPs and regulating CheY~P hydrolysis [78,79]. The presence of genes with unknown function within chemotaxis operons has been reported in various bacteria, and appears to be commonplace in bacteria with more complex chemotaxis pathways [27,80-82].

Based on these observations, we suggest that the Type 1 *che *clusters functions like the *E. coli *chemotaxis pathway, albeit with the differences noted above, in which case *che *cluster 1 of *G. metallireducens *regulates signaling through a lone class 36 H MCP (*Gmet1078*) [34]. If cluster 2 of *G. uraniireducens *serves a similar role, then a lone 36 H MCP (*Gura2167*) serves to detect the environmental stimuli in this situation as well. According to this reasoning, we do not expect *G. sulfurreducens *PCA (AE017180.1) to have a flagellar-based chemotaxis pathway that uses this signaling logic, because it lacks both a Type1 *E. coli*-like *che *gene cluster and class 36 H MCPs. However, the absence of class 36 H MCPs does not rule out other modes of flagellar-based motility or chemotaxis. For example, the chemotaxis pathway in *B. subtilis *uses class 44 H MCPs, and the genome of *G. sulfurreducens *contains several *mcp *genes that belong to this class. Fewer investigations of Type 2 *E. coli*-like *che *clusters have been conducted, yet in their study of a Type 2-like cluster in *R. sphaeroides*, Armitage and colleagues found that this cluster is essential for flagellar-based motility [83]. All the *Geobacter *sp. genomes contain at least one Type 2 cluster; these too could potentially participate in flagellar-based chemotaxis. Further work is needed to verify the actual functions and relationships of Type 1 and 2 *E. coli*-like clusters, which will serve strengthen the confidence of predictions based on percent gene identity, gene cluster organization and mechanistic similarities reflected in protein organization.

### Dif-like Clusters May Regulate Extracellular Matrix Formation and Chemotactic Motility

*G. sulfurreducens *and *G. uraniireducens *possess clusters comprised of similar genes and gene ordering to the *dif *cluster of *M. xanthus*. These clusters contain class 44 H MCPs with two predicted transmembrane segments, but small periplasmic domains (~3 to 10 aa), genes for CheA, CheW, CheY, CheC and CheD, and genes with unidentified function. (Figure S4 lists the gene arrangements and percent identities of the individual gene products [Additional file 1].) The *dif *signaling system of *M. xanthus *has been studied most, where it is known to be involved in the regulation of exopolysaccharide formation, an essential component of the *Myxococcus *social motility apparatus [61,84]. It has been noted that phenomenon of social motility in *M. xanthus *resembles biofilm formation in other bacteria [85]. In addition, the *dif *cluster is also involved in sensing of certain lipids [24]. One difference between the *M. xanthus dif *cluster and the *Geobacter dif*-like clusters is the presence of *CheR *in the *Geobacter *cluster instead of *difB*. A plausible consequence of this observation is that the *Geobacter dif*-like pathways are CheR-dependent, whereas the *M. xanthus dif *system is CheR-independent. The involvement of *Geobacter dif*-like clusters in the synthesis of extracellular matrix material, which is essential for biofilm formation, is currently under investigation.

### Che Clusters with CheA/Y Fusion Proteins

*G. metallireducens *and *G. uraniireducens *each have one *che *cluster with a gene that encodes a CheA-CheY fusion protein (CheA/Y). In *R. centenum*, *M. xanthus, P. aeruginosa*, and *Synechocystis *strain PCC6803, *CheA/Y*-containing *che *clusters carry out various functions, including the regulation of flagellar-based motility [86,87], typeIV-pili based motility and/or the biosynthesis of typeIV pili [21,25,26], cell development [17,23], and biofilm formation [16]. The *Geobacter che *clusters in Figure 1 that encode CheA/Y fusion proteins are most similar to the *M. xanthus *Frz cluster, cluster 3 of *P. aeruginosa*, and cluster 3 of *R. centenum*. This conclusion was reached through comparisons of the gene cluster content, gene order and the percent identity among CheA/Y homologs (Figure S5) [see Additional file 1]. These gene clusters function in developmental cell aggregation [88], biofilm formation [16], and cyst cell development [17], respectively – processes that involve cell-cell interaction. By these same criteria, the *Geobacter *clusters were least similar to *che *cluster 1 of *R. centenum *(chemotactic and photactic responses [86]), *M. xanthus *cluster 3 (the regulation of fruiting body formation [23]) and *Synechocystis *cluster 2 [26]. Overall, these findings suggest that the corresponding *Geobacter che *clusters may also regulate processes involving cell-cell interactions and/or social motility, but this idea is in need of experimental proof.

### Che Clusters that are Unique to the Geobacter Species and δ-Proteobacteria

Two groups of *che *clusters are highly conserved among the *Geobacter *sp., we refer to these as α and β groups. The clusters belonging to these two groups contain the well-known homologs of chemotaxis genes (*cheA*, *cheW*, *cheB*, and *CheR*), but no *mcp *genes. Cluster 1 of *G. sulfurreducens *and cluster 7 of *G. metallireducens *and *G. uraniireducens *belong to the α group; the β group *che *clusters are 5, 2 and 5, respectively (Figure 1). An extensive search of both completed and draft bacterial genomes led to the finding that group α *che *gene clusters are present only in the genomes of the *Geobacteraceae*, including *Geobacter bemidjiensis *Bem ctg130, *Geobacter lovleyi *SZ, *Pelobacter propionicus *DSM 2379, and *Geobacter sp*. FRC-32.

Group α clusters have not been found in genomes outside the *Geobacteraceae *family. (See Figure S6 [Additional file 1] for gene arrangements and percent identities.) Each group α cluster contains a gene encoding a protein with an HD domain – which defines membership in an enzyme superfamily of metal-dependent phosphohydrolases, where the conserved His-Asp (HD) doublet has a role in catalysis [89]. Within a variety of contexts, HD-domain-containing proteins have diverse biochemical functions, including nucleic acid metabolism and signal transduction. The predicted *Geobacter *homologs contain no other recognizable domains, *i.e*. they may function as standalone proteins. Standalone HD domain proteins in *E. coli *have low amino acid identity with each other and to the *Geobacter *homologs (~10%), yet the *E. coli *proteins all act on nucleotide substrates [90]. The predicted HD domain proteins located within the group α *che *clusters are probably regulated by, or participate in, chemotaxis-like signaling pathways of special significance to the cellular physiology of *Geobacter*.

Group β clusters are conserved among δ-*Proteobacteria*, and have been detected in the genomes of *G. bemidjiensis*, G.*lovleyi*,*Geobacter sp*. FRC-32, *Stigmatella aurantiaca *DW4/3-1, *Anaeromyxobacter dehalogenans *2CP-C, *Plesiocystis pacifica *SIR-1, and *Myxococcus xanthus *DK 1622. (See Figure S6 [Additional file 1] for gene arrangements and percent identities.) In general, the functions of β group clusters are not known. However, we have obtained preliminary results with *G. sulfurreducens *cluster 5 knockout mutants, which indicate that this β group cluster regulates the expression of extracellular matrix material, and may represent a new way that chemotaxis-like signaling pathways can participate in biofilm formation (HT Tran, DR Lovley and RM Weis, *unpublished observations*).

### Chemotaxis Gene Expression Regulated by Alternative Sigma Factors 28 and 54

The mechanisms for regulating the expression of chemotaxis and flagellar genes are complex, but diverse, and should provide clues to the diversity and purpose of chemotaxis-like signaling systems. Therefore, we conducted a preliminary investigation into the regulation of *che *gene expression, in particular σ^28^- and σ^54^-regulated promoters upstream of *che *and flagellar gene clusters. In *E. coli *and *Salmonella*, the *che *and late flagellar genes, including *fliC *(the flagellar filament), are positively regulated by σ^28 ^[91-93]. In other bacteria, especially those with more than one *che *cluster, expression is also regulated by σ^54 ^[94]. For instance, *R. sphaeroides *has a σ^28^-regulated system that shows coupled expression of the chemotaxis proteins and flagella, and a system that regulates flagellar synthesis independently *via σ *^54 ^[95].

**Figure 4:**
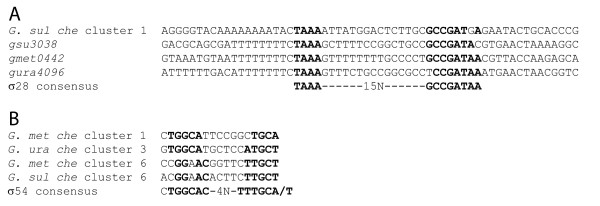
**Putative σ^28 ^and σ^54 ^promoter elements**. (A) Putative σ^28 ^promoter regulation sites found upstream of *G. sulfurreducens che *cluster 1 and the *fliC *genes of *G. sulfurreducens *(*gsu*3038), *G. metallireducens *(*gsu*0442), and *G. uraniireducens *(*gsu*4096) [97]. 125, 160, 127, and 152 nucleotide bases separate the predicted transcription start sites from the start codons, respectively. (B) Putative σ^54 ^promoter elements upstream of the *Geobacter *major *che *gene clusters [51]. For *G. metallireducens *clusters 1 and 6, *G. uraniireducens *cluster 3, and *G. sulfurreducens *cluster 6, the predicted transcription start sites are 50, 33, 24 and 16 nucleotide bases upstream of predicted operon ATG start codons, respectively.

We searched upstream of the major *Geobacter che *operons and *fliC *loci for evidence of σ^28^-regulated expression. As Figure 4A shows, σ^28 ^binding sites were identified upstream of *fliC *in *G. metallireducens*, *G. sulfurreducens *and *G. uraniireducens*, but only one major *che *cluster, the *G. sulfurreducens *group α cluster (cluster 1, Figure 1), had a recognizable σ^28 ^binding site. Therefore, it seems that the specific mechanisms of regulation for most of the *Geobacter che *clusters will be different from *E. coli *(and *Salmonella*).

The *G. sulfurreducens *genome was searched for σ^54 ^recognition sites to determine the number of *che *gene-related sites relative to all the sites that may exhibit σ^54 ^regulation. Of the 110 sites identified genome-wide, nine were located in noncoding regions upstream of *che*, *mcp *or flagellar operons (Figure S7 lists positions and sequences of the chemotaxis-related promoter sites [Additional file 1]) – one of these was the *Dif*-like cluster (cluster 6, Figure 1). Focused searches upstream of the major *che *clusters in the other two species identified possible σ^54^-regulated promoter sites before cluster 3 in *G. uraniireducens*, and clusters 1 and 6 in *G. metallireducens *(Figure 4B). No correlation was apparent between the identity of these clusters and their mode of regulation, *i.e. G. metallireducens *cluster 3 is classified as an *E. coli*-like cluster, and the other two do not belong to any identified class. Consequently, little specific insight can be gleaned from these early findings. Nonetheless, the results may presage a diversity of mechanisms for regulating expression. Indeed, we can expect that once the *che*-gene-specific regulatory elements are known (which is significant in itself), it will be a challenge to determine how these systems map onto the global patterns of gene expression; this pattern should reflect how *Geobacter *adapts to the complex environment it inhabits.

## Conclusion

The comparative analysis of *che *gene clusters and regulatory sequences among *Geobacter *sp. and to other bacteria has provided valuable insight into the functions of the various *Geobacter *chemotaxis-like pathways. The genomes of *Geobacter *sp. have multiple copies of chemotaxis genes – more than 60 per genome. Their arrangement in six to seven major clusters reflects both greater complexity and diversity in comparison to the single cluster on the *E. coli *chromosome. This diversity is also reflected in the presence of both σ^54^- and σ^28^-dependent regulatory sequences. The presence of multiple chemotaxis-like clusters and mechanisms of regulation both suggest that the pathways in *Geobacter *are not redundant, but instead each fills a specific cellular need.

*Geobacter *species have several clusters in addition to a *che *cluster that is similar in organization to the chemotaxis operons of *E. coli *and *S. enterica*. These clusters are similar to known clusters in other bacteria that regulate functions other than flagellar-dependent motility. From our analysis, it seems probable that *Geobacter *sp. use chemotaxis-like signaling pathways for a variety of functions, which probably include type IV pili-based motility, regulation of motility apparatus expression (flagellar, pili, and extracellular matrix), and biofilm formation. Interestingly, the *Geobacter *sp. have *che *clusters that – at the present time – appear to be unique, which may plausibly mean these pathways regulate physiological functions that are unique to the *Geobacters*. Sensory inputs to the chemotaxis-like pathways are likely to be diverse, because the *Geobacter *genomes contain a large number of chemoreceptor (*mcp*) genes, which display a diversity of sensing domain architecture. The presence of this large number of proteins – receptors and Che proteins – undoubtedly reflects a greater need for the *Geobacters *to respond to a variety of environmental conditions, which allows them to thrive in subsurface environments. The presence of MCPs that belong to different MA domain classes in one genome – *i.e*. express MCPs in the same cell membrane with MA domains of different lengths, may contribute to the segregation of receptors into class-specific clusters with their cognate Che signaling proteins. We postulate that this mechanism will generate pathway specificity and diminish unwanted cross-talk. Such a mechanism can be general for bacteria with multiple chemotaxis-like pathways.

## Abbreviations

MA: Methyl-accepting; MCP: Methyl-accepting chemotaxis protein.

## Authors' contributions

HT and RMW developed the main concepts and drafted the manuscript. HT carried out the sequence alignments, and homology and phylogenetic tree analyses. JK directed computational analyses of operon predictions and σ^54 ^promoter regulation. FA participated in the computational analysis of the MCPs. RMW, JK, and DRL read and provided feedback on the manuscript.

## Supplementary Material

Additional file 1 **The file contains the additional information figures and tables.** Click here for file

Additional file 2 **The file contains the multiple sequences alignment of *Geobacter* mcp genes.** Click here for file
