# Supplementary material for: Comparative genomics of Geobacter chemotaxis genes reveals diverse signaling function
Source: BMC Genomics. 2008 Oct 9;9:471. doi: 10.1186/1471-2164-9-471 (PMC2577667; doi:10.1186/1471-2164-9-471)
Supplement: Additional file 1 — The file contains the additional information figures and tables. [file 1471-2164-9-471-S1.pdf]

Supplementary Figures and Table

Comparative Genomics of *Geobacter* Chemotaxis Genes Reveals Diverse Signaling Functions

Hoa T. Tran, Julia Krushkal, Frances M. Antommattei, Derek R. Lovley and Robert M. Weis

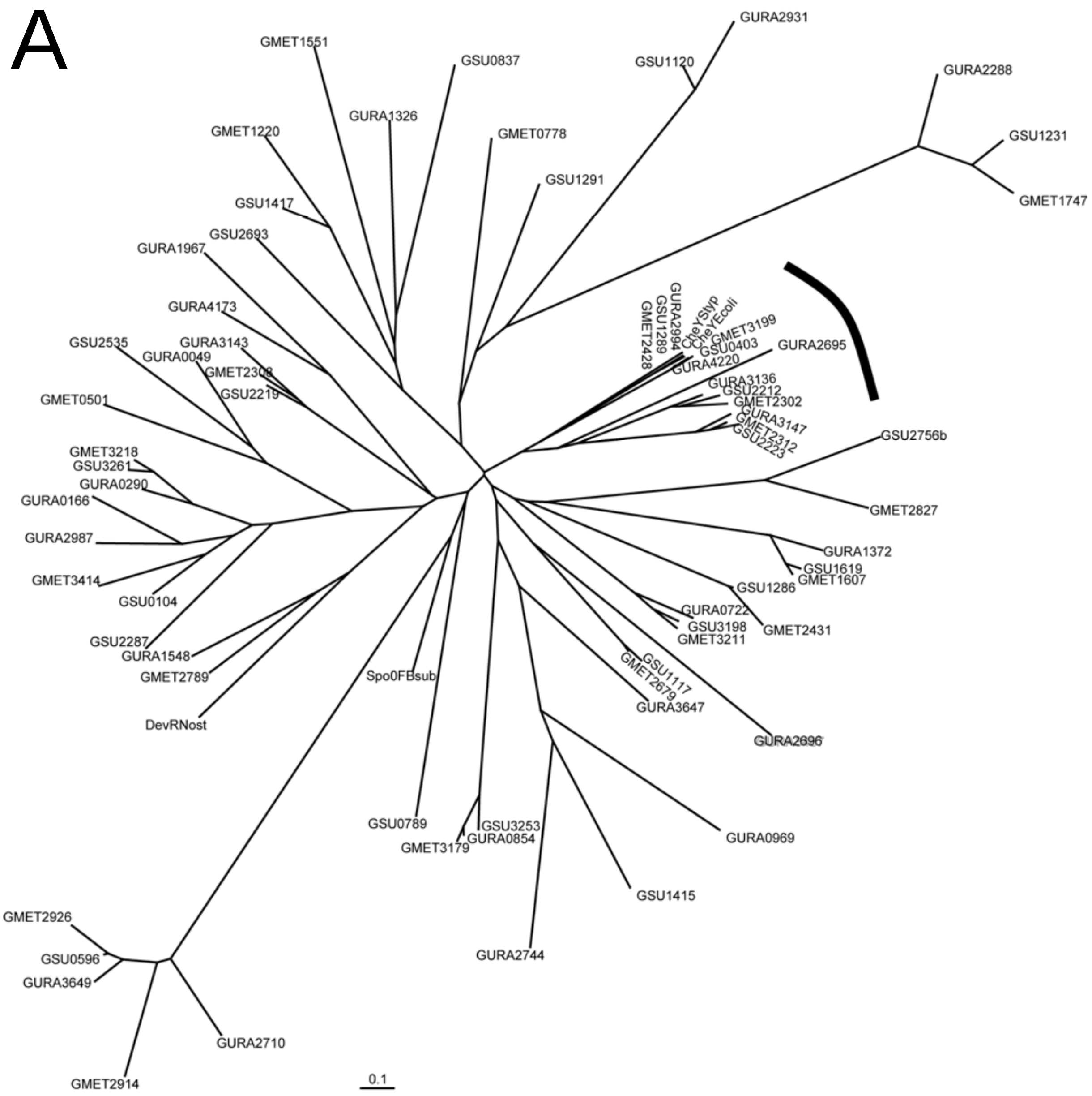

B	***Geobacter* CheY Homologs**			
	***cheY*s in *che* and *fla* clusters**		***cheY*s outside *che* and *fla* clusters**	
**Species**	***E. coli* Branch**	**Other Branches**	***E. coli* Branch**	**Other Branches**
G. sul	4/7	3/7	0/15	18/18
G. met	4/10	6/10	0/15	11/11
G. ura	5/10	5/10	0/15	15/15

Figure S1. The *Geobacter* CheY-like Phylogenetic Tree (A) and the Homolog Distribution within the Tree (B). (A) A neighbor-joining phylogenetic tree of the *Geobacter* CheY homologs and selected CheY & CheY-like proteins from other species: *E. coli* (Ecoli), *Bacillus subtilis* (Bsub), *Salmonella enterica* subsp. enterica serovar Typhi Ty2 (Styp), and *Nostoc* sp. strain ATCC 29133 (Nost). **(B)** *Geobacter* CheY homolog distributions between the tree branch with *E. coli* CheY and the rest of the phylogenetic tree. ~50% of the homologs situated on the chromosome within the major *che* or flagellar gene clusters reside in the tree branch that contains the *E. coli* and *S. typh* CheYs (marked by a black arc). No CheY homologs situated on the chromosome outside *che* or flagellar gene clusters reside in the same branch with *E. coli* CheY.

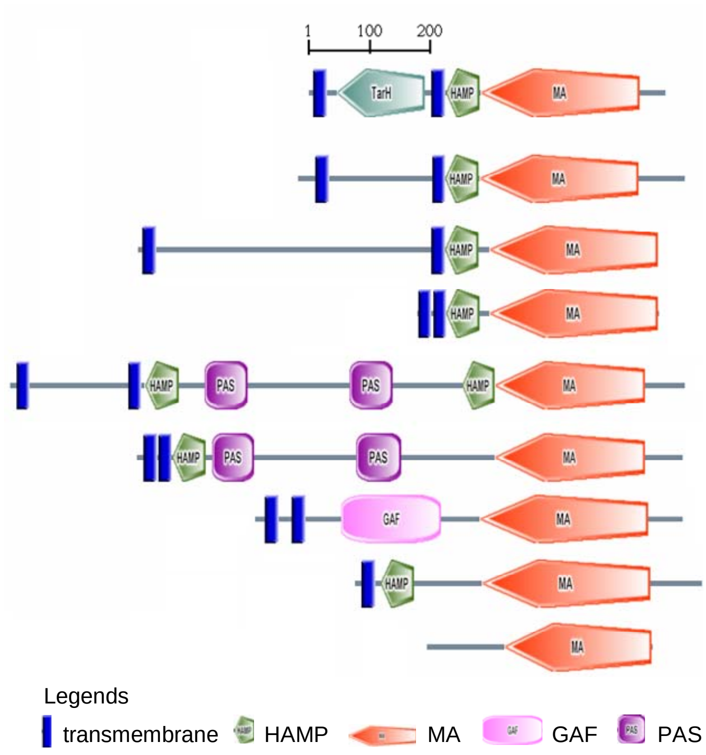

G. met (18)	*G. sul* (34)	*G.ura* (24)	
			E.Coli Tar
11	25	15	2TMs, regular periplsam (~150-200 aa)
1	1	1	2TMs, large periplasm (~350-450 aa)
2	3	4	2TMs, small periplasm (<20 – (~100 aa))
1	0	1	2TM, regular periplasm and PAS domain
0	0	1	Aer like
1	1	0	2TMs, GAF domain
0	1	0	1 TM
2	3	2	Soluble

Figure S2. Domain architectures of MCPs in *Geobacter* sp. show diversity. *Geobacter* MCPs are classified according to the periplasmic domain size, the length of the methyl-accepting (MA) domain [1] and the presence of the PAS [2] (*gmet2422*, *gura1191*, *gura2989*) or the GAF domain [3] (*gsu1704*, *gmet1641*).

Table S1. Distribution of *Geobacter* MCPs by number of transmembrane segments & size of periplasmic domains.

Number of Transmembrane Helices	Size of Periplasmic Domain (amino acid residues)	MCP of Each Type (%)		
		G.sul	***G.met***	***G.ura***
**2**	Large (250 to 430)	3	0	4
	Medium (150 to 200)	**76**	**72**	**67**
	Small (< 100)	9	17	21
**1**		3	0	0
**0 (Soluble)**		9	11	8

Figure S3. Gene arrangement of *E. coli*-like clusters in *Geobacter* (A, C), with the percent identity of the individual gene products to *E. coli* (B, D)

A. Type1 gene arrangements

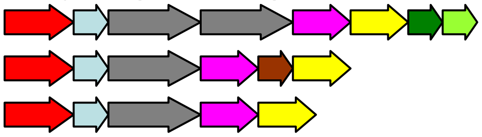

E. coli

Cluster 1 *G. metallireducens*

Cluster 2 *G. uraniireducens*

B. Percent identities of the *Geobacter* Type 1 cluster proteins with *E. coli*. The MCP identities were determined between the *E. coli* aspartate receptor cytoplasmic domain (c-Tar) and the corresponding c-domains of *Geobacter* MCPs. For CheA, CheW, CheR and CheB the full length gene products were used.

E. coli	**CheA**	**CheW**	**Tar Cyt. Domain (c-Tar)**	**CheR**	**CheB**
G. met (cluster 1)	47	63	53	50	62
G. ura (cluster 2)	46	60	53	48	60
**Mean identities with remaining *Geobacter* homologs**[a]					
G. met	32 ± 5 (4)	27 ± 5 (8)	29 ± 6 (7)	29 ± 5 (5)	37 ± 8 (5)
G. ura	32 ± 3 (6)	27 ± 4 (6)	27 ± 6 (7)	32 ± 4 (7)	41 ± 7 (5)

[a]mean ± standard deviation (number of homologs in the average); including Type 2 clusters.

C. Type 2 gene arrangements

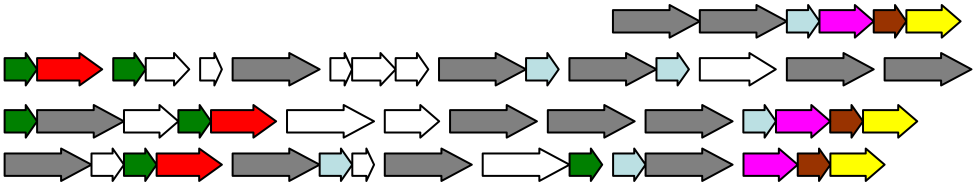

Cluster 3 *G. sulfurreducens*

Cluster 4 *G. sulfurreducens*

Cluster 3 *G. metallireducens*

Cluster 4 *G. uraniireducens*

D. Percent identities determined between the gene products of Type 2 clusters and *E. coli* proteins (as described in S3 B). For CheW and CheY, the percent identities of both loci are reported. For the MCPs, the mean and standard deviation of the identities are reported, and the number of sequences used is in parentheses.

E. coli	**CheA**	**CheW**	**c-Tar**[a]	**CheR**	**CheB**	**CheY**
G. sul (clusters 3 & 4)	37	33 ± 1.5 (3)	35 ± 3 (7)	41	50	26, 37
G. met (cluster 3)	36	31	33 ± 1 (3)<br>15 (1)	38	50	23, 37
G. ura (cluster 4)	35	29, 30	32 ± 1 (3)<br>14 (1)	37	50	25, 35
**Mean identities with remaining *Geobacter* homologs**[b]						
G. sul	32 ± 2 (3)	27 ± 7 (6)	25 (1)	29 ± 5 (3)	38 (2)	29 ± 3 (4)
G. met	30 ± 6 (3)	27 ± 4 (5)	29 ± 1 (3)	26 ± 7 (4)	35 ± 3 (4)	29 ± 5 (5)
G. ura	32 ± 4 (5)	26 ± 5 (6)	27 ± 2 (3)	27 ± 6 (6)	37 ± 3 (4)	26 ± 7 (6)

[a]mean ± standard deviation (number of homologs in average). One c-domain of lower identity, as noted, was excluded from the average.
[b]mean ± standard deviation (number of homologs in average); excluding Type 1 clusters.

Figure S4. Gene arrangement of the *dif*-like clusters in *Geobacter* species (A) and the percent identity of the individual gene products in the cluster (B)

A. Gene arrangement

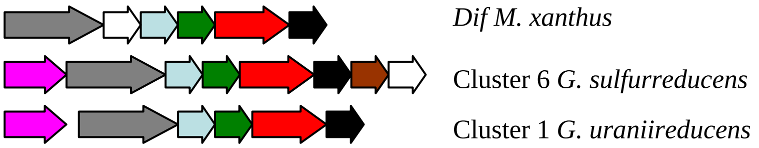

B. Percent identities of full-length gene products in the *Geobacter dif*-like clusters to *M. xanthus dif*-homolog proteins. (The *E. coli* homologs are indicated in parentheses; c-DifA refers to the c-domain of the *M. xanthus* MCP.)

M. Xanthus	**c-DifA (MCP)**	**DifB**	**DifC (CheW)**	**DifD (CheY)**	**DifE (CheA)**	**DifG (CheC)**
G. sul (clust. 6)	38	No homolog	18	54	43	33
G. ura (clust. 1)	38	No homolog	21	52	41	31
**Mean identities with remaining *Geobacter* clusters**[a]						
G. sul	27 ± 3 (7)		17 ± 2 (6)	28 ± 4 (5)	28 ± 4 (3)	No homolog
G. ura	23 ± 4 (5)		16 ± 4 (7)	24 ± 6 (8)	28 ± 4 (6)	No homolog

[a]mean ± standard deviation (number of homologs in average).

Figure S5. Gene arrangements in *Geobacter* clusters containing *cheA/Y* fusions compared to other species whose functions have been identified (A), and the percent identities of *cheA/Y* fusions (B)

A. Gene arrangement

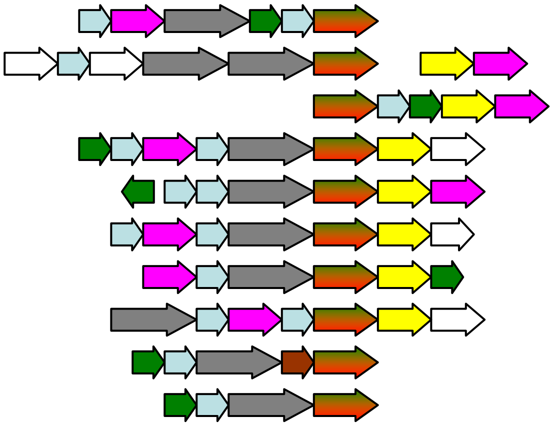

B. Percent identities between *Geobacter* CheAY fusions with fusions in other species.

		**Identity (%)**	
**Species and Cluster**	**Protein Name**	***Gura*4171**	***Gmet*2710**
M. xanthus Frz Cluster	FrzE	33	28
P. aeruginosa Cluster 3	WspE	30	29
R. centenum Cluster 3	CheA3	30	32
Synechocystis Cluster 1	TaxAY1	30	27
R. centenum Cluster 1	CheA1	29	26
M. xanthus Cluster 3	CheA3	27	29
Synechocystis Cluster 2	TaxAY2	25	24

Figure S6. Gene arrangement of other *che* clusters

Group α: *che* cluster conserved among *Geobacteraceae*

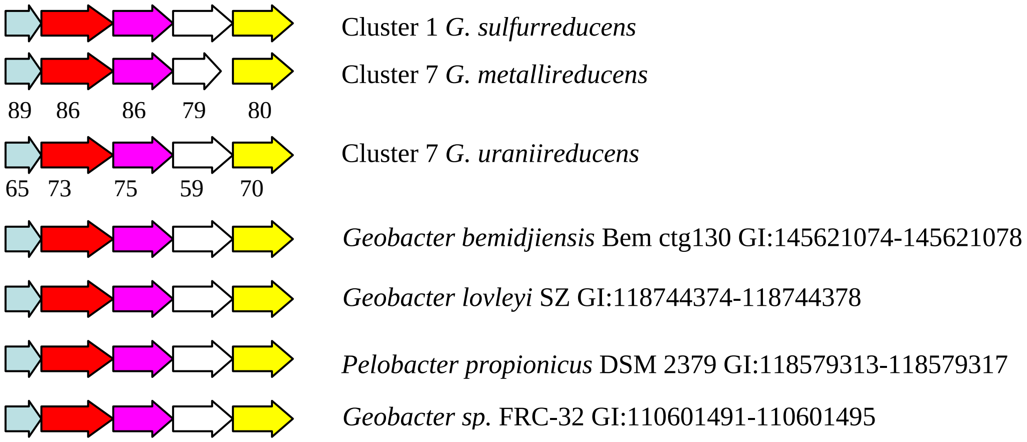

Percent identities of the full-length *G. metallireducens* and *G. uraniireducens* gene products compared to *G. sulfurreducens* are shown below the *metallireducens* and *uraniireducens* clusters. Clusters found in other *Geobacteraceae* are shown with the corresponding NCBI Gene Identifier numbers or locus tags on the right.

Group β: *che* cluster conserved among *delta-proteobacteria*

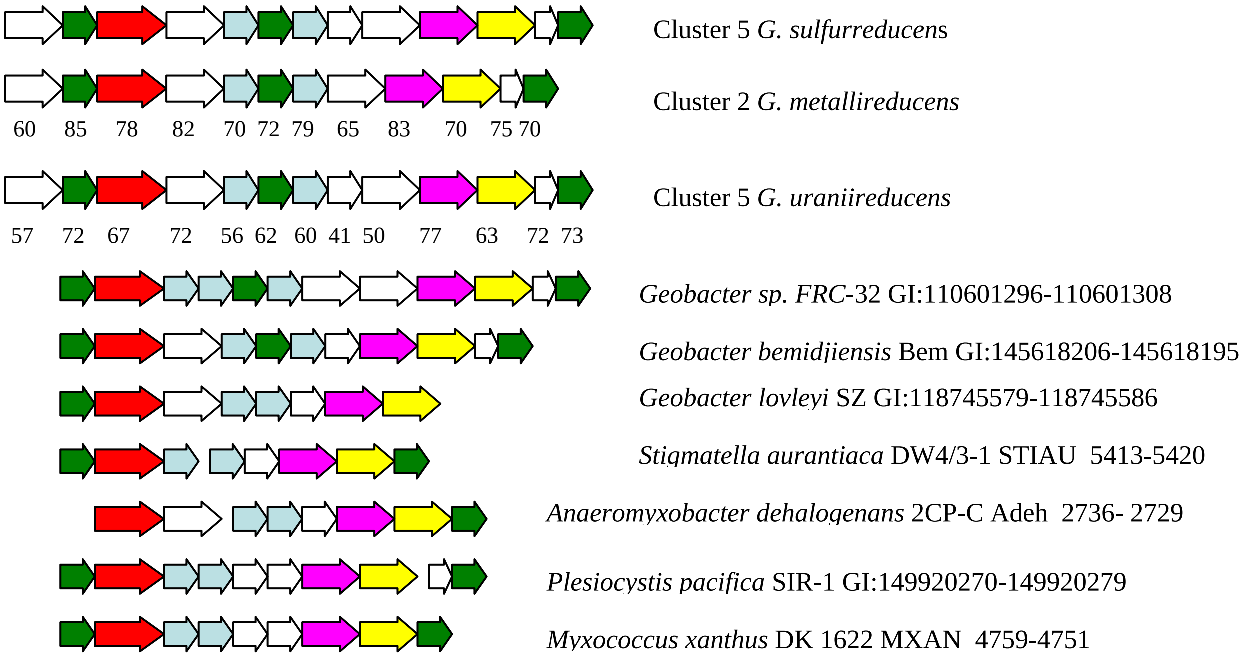

Numbers below each gene are percent identities of the full-length *G. metallireducens* and *G. uraniireducens* gene products compared to *G. sulfurreducens*. The clusters found in other δ-*proteobacteria* are shown with the corresponding NCBI Gene Identifier numbers or locus tags on the right.

Figure S7. Chromosome locations of σ^{54} regulation elements in *G. sulfurreducens* upstream of *che* and flagellar gene operons

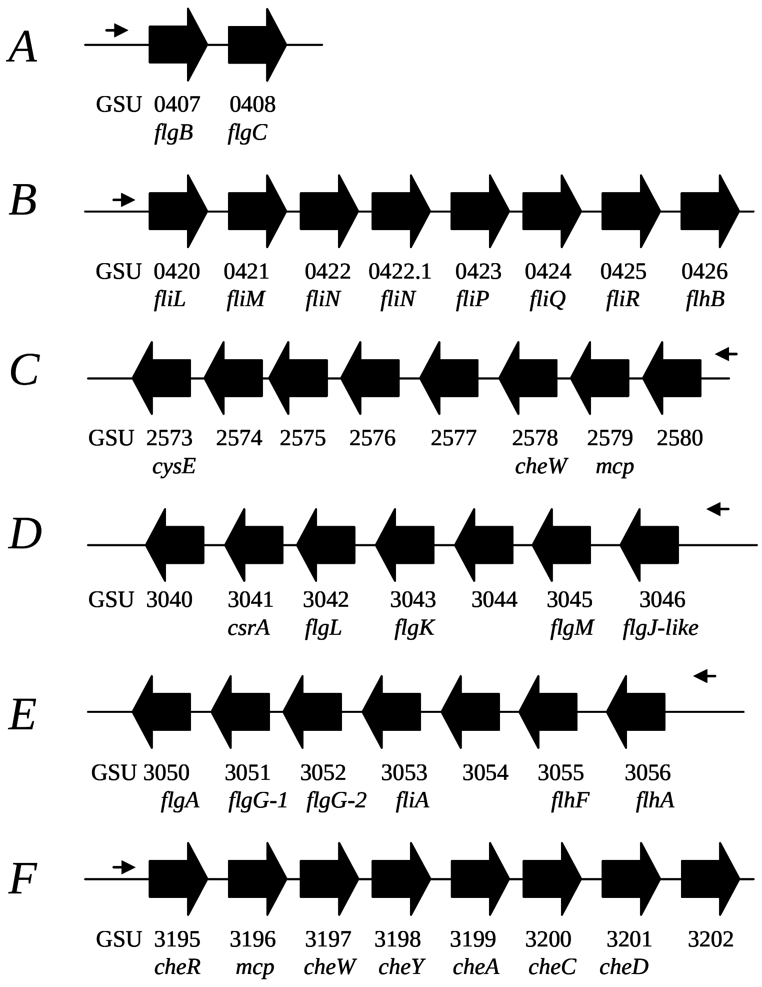

Fig.	σ^{54} sequence	Score	Position	Operon	Bases from 1st ATG
A	CTGGTACGGCTTTTGCT	92	439442-439458	*Gsu*0407-0408	23
B	CTGGCATTTCGGTTGCA	86	451031-451047	*Gsu*0420-0426	47
	ATGGCACGGCCTGTGTA	81	773999-774015	*Gsu*0725-0726	26
C	TTGGCATCCTGCCTGCT	81	2842557-2842541	*Gsu*2573-2580	48
	TCGGCACGTAGGTTGCA	86	3311556-3311572	*Gsu*3017-3028	48
D	TTGGCACATAACATGCT	86	3350142-3350126	*Gsu*3040-3046	48
E	CTGGCACAACGGTTGCA	93	3359815-3359799	*Gsu*3050-3056	39
	ACGGCACCGGCATTGCC	80	3462928-3462944	*Gsu*3156 (*mcp*)	115
*F**	ACGGAACACTTCTTGCT	81	3503117-3503133	*Gsu*3195-3202*	16
	**CTGGCAC**-4N-**TTTGCA/T** (*consensus*)				

*****Gsu*3195-3202 is *G. sulfurreducens che* gene cluster 6 (Figure 1).

Supporting Materials References

1. LeMoual H, Quang T, Koshland DE: **Methylation of the Escherichia coli chemotaxis receptors: Intra- and interdimer mechanisms.** *Biochemistry* 1997, **36:**13441-13448.
2. Ponting CP, Aravind L: **PAS: a multifunctional domain family comes to light.** *Curr Biol* 1997, **7:**R674-677.
3. Aravind L, Ponting CP: **The GAF domain: an evolutionary link between diverse phototransducing proteins.** *Trends Biochem Sci* 1997, **22:**458-459.
