# Supplementary material for: Comparative genomics of Geobacter chemotaxis genes reveals diverse signaling function
Source: BMC Genomics. 2008 Oct 9;9:471. doi: 10.1186/1471-2164-9-471 (PMC2577667; doi:10.1186/1471-2164-9-471)
Supplement: Additional file 2 — The file contains the multiple sequences alignment of Geobacter mcp genes. [file 1471-2164-9-471-S2.html]

--------HAMP
/--------------------------------------------------------------------------------------------------------------------------------------/
Highly conserved domain
/----------------------------------------------------


```
               --------HAMP   /--------------------------------------------------------------------------------------------------------------------------------------/      Highly conserved domain      /--------------------------------------------------------------------------------------------------------------------------------------------------------------------------------------------------------------------------------------------------------------------------------------------------------------------
```

```
GSU2372        ETSSSILEMAASIEEVAVN-----------VDSLAQAVDEVSSSVMEMAASIKQIANSVVSLQDVTTTTASSVAEMDSSIRQVEKNAMETASISEGVRRDAE-MGKVSVEATIAGINEIKRSSRITSEVIETLSVRATDIGAILSVIDEVAEQTNLLALNAAIIAAQAGEHGKGFAVVADEIKELAERTTSSTREIAQLIKGVQDETARAVEAIELAEKSIADGEALSQKSGEALAKIVTGVQGATAQVESIARATMEQAKGSQMIRSAMERVSDMIAQVAGATREQGKGSDM--------------IMAAAERMKGLTSQVRTSTREQSKVGAFIARSTENITDMIQQIKRACDEQSRGSDQIIRAVEDIQESTSTNLGSARMMDDAVSRLSRQLEALERGMSSFKVENR------------------------------------------------------------------------------------
```

```
Gmet2478       ETSSSILEMAASIEEVALN-----------AEALAQSVEEVSSSVVEMVASIKQISGSVASLMEATSSTASSVAEMDSSIKQVEKNAMESAAISEGVRRDAE-TGKASVEATIAGISEIKRSSRITSEVIETLSERVSDIGAILSVIDEVAEQTNLLALNAAIIAAQAGEHGKGFAVVADEIKELAERTSSSTREISLLIKGVQDETARAVEAIEVAEKSIADGEILSQRSGEALAKIVTGVQETTAQVESIARATMEQAKGSQMIREAMEQVSDMIGQIASATREQSKGSDM--------------IMGAAERMKGLTSQVRVSTKEQAKVGNFIASSTENITTMIRQIKRACDEQTRGSEQIIRAVENIQESTDTNLGAAKMMEDSVSRLSRQLEVLQGEMNSFKVENQGAVPKG------------------------------------------------------------------------------
```

```
Gura3281       ESSSSILEMAASVEEVAQN-----------VETLSHSVDEVSSSILQMAASIKQVGNGVVSLLDASTTTASSVMEMDSSIKQVEKNAMETAAISDAVRKDAE-TGKEAVEATIAGINEIKRSSRITYEVIDTLSGRANDIGTILSVIDEVAEQTNLLALNAAIIAAQAGEHGKGFAVVADEIKELAERTSSSTREIAMVIKAVQDETHRAVNAIDQAEKSIADGELLSQKSGEALNKIVSGVKKATEQVEEIARATVEQARGSQMIRDAMEQVSEMVGQIAKATREQGQGSEL--------------IMTAVEKMKTLTTQVRSSTREQSNVGNFIAQSTENITDMIQQIKRACDEQNRGSEQIVVAVEDIQQSTHINLEATGAMDEAVSGLFRQIEILRNEMGMFKIEG-------------------------------------------------------------------------------------
```

```
GSU0583        ENAAAIVQMSTSIEVVAEH-----------MEGLAREVDEVSSSIIQMAAAEKEIGRSVRVLMEDASRTASLVAEMDLSIRQVEKSALETAAISEEVLRDAE-LGRDSVDRTISGISEIRRSSRSASDTITTLSHRVGDIGTIISVINEIAEQTKLLALNASIIAAQAGEHGKGFAVVANEIKELAKRTTSSTGEIAEIISGLREETVRAVQAIKQAEDRIGEGETLSYRSGEALEKIVDGVKMAVDQVGEIARTTVEQAQGSENMRRAMERVAEMVEQIMRATQEQAHGTEL--------------ITEAADRMKSLTGRVFSSTREQRDTSTHIVRSSEGVTHMISTIRQASQVQAENSQKIVEAVENMETTAVNGLDTTRLMEEAVSRLARQTEGLTEAMAGFKVR--------------------------------------------------------------------------------------
```

```
Gura0165       ENSSSILEMSASIEEVIKH-----------VEALANAVEEVSTSISEMAAAEKQIGASVNNLMADSTTTAKLVAEMDGSIKQVERNALNTATISEEVRNDAE-SGRVSVEATISGIGEIRRSSRITFEAIQNLSLRAGNIGKIILVIDELAEQTNLLALNASIIAAQAGEHGKGFAVVAEEIKELARRTGNSTREITDIIKGVQEETQRAVKAINLSEQRIVEGEQLSQRSGEALNKIVAGVQMATDQVSQIARTTVEQAQGSQEISRAMERVADMVKQIAKATREQGHGSEL--------------IMSAVERMKGLTSQVRSSTLEQSSSSNLIVRSTEDITTMIMNIRQACTVQTESSRQIVAAIEHIQQSTKTNVESTRVMDGAMAGLSRQIEVLSNEMSDFKV---------------------------------------------------------------------------------------
```

```
GSU0766        DTSSSILEMTASVEEVAIN-----------ADNLSRLVDEVSSSVIQMAASIKQIDGSVQSLMEISTTTASSVAQMDTAIGQVEVNARETTALSQDVEREAE-RGKRAVEDAIAGIVAIQRSSRITTEVIDVLSRKVEDIGGIISVIDEIAEQTNLLSLNAAIIAAQAGEHGRGFAVVAGEIKDLSDRTRTSTREIAEVIMGVQSETRRAVEAISRADKSIADEERLSANADEALGKIVMRAREASSRVAEIARATVEQATGSKIIRDAINRVTDMTSQIASATSEQGAGGDL--------------IMTAVERMRDATAQVRNSTREQSATGNIIARSTENITDMIGQFRSASEEQFRGSEQIVRSMEEIQQSATMSLEVSRVMEEAAITLSRQVKVLETEMEGFHIRGQSSSR--------------------------------------------------------------------------------
```

```
Gura0349       ESSSSILEMTASVTEVAHN-----------AETLNKSVGEVSSSIVQMTASIKRVGSSVGNLQEAAASTSSSVMQMDTSIKQVERSAAAAAAISDEVRGDAE-FGRSAVEASIVGISEIKRASDITSEVINSLSERASAIGVILSVIDEVAGQTNLLALNAAIIAAQAGEHGKGFAVVADEIKELAERTSASTQEITKVISAVQNETARAVEAIHIAEKSIADGKDLSEKSGEALKKIYEGVQKATDQMREIALTTLEQSKGSQMIREAMEQVSEMVGQIAKATKEQSQGSEL--------------IMSSAEHMKQLTEQVRNSTLEQSKVANFIAQSTENITSMISTISRASGEQSRGSDQIAHAVEDIQSSASINLDATKVMDKVVTNLFMQIGVLREQMKVFKV---------------------------------------------------------------------------------------
```

```
Gura4018       ESSSSVLELAESIETVARN-----------MENLATSVDGISVSIIQMTESIKQIDAGVQALTDTSTSTASSVLEFDTSIRQIEAYAKESAAISDAVRCDAE-TGKKAVDETIVGIDGITHASRVAAEAIGSLSQKAQSIGSIITVIDEIARQTNLLALNASIIAAQTGAHGKGFGVVAAEIKQLAERTTRSTREIAEAITGVQSETSRAVSAIAAAEESIKTGEQLSLQAGNTLGKIVEGVDRTAVQMAEIARATREQAKGSELIRTAMEQVATMANSIADTTRQQRKGSEL--------------IHTEVGRVREFSSMVMRSMKEQATVGESISRMTLHVSESSSRIREACVDQTNGSLRIQTVVESIQRSTSTVRQETRVVDNGVSKLGANTESLQNEMANFTL---------------------------------------------------------------------------------------
```

```
GSU1704        NLKDAVNSVADGTAHIASA-----------SESVLSAVDETSSAVSNIYVAIEQVTRNIDYLSESIDKSVSAMEELNSSIKNVEQSAAISHQVSSTVKEKAD-SGRAVVDETIQALDEIQRSVDQSFEAMKRLSENSGKIESIVGVINDITKRTNLLALNASIIAAQAGEYGKSFGVVADEIRNLSLQTGQSTGEITGIIEEIMRESRSAAQNITASKDLVQRGVELGGIMGQSLQVIHESSTRSMDMTHEIKTATEEQARSVQLVTNSIENVSSMSTQIYKASKEQSDAAMS--------------IVRSVDTIKEMAQEMVRATVKQVEDGSEIKKSVEAVGEMVTRIFEDMEVRREESGEVVKELELMKKIAS-----------------------------------------------------------------------------------------------------------------------
```

```
Gmet1641       NLKDAVNSVADGTAHIASA-----------AESVLASVDETSSSVSNIYVSIEQVTKNLDYLSESIEKSVSAMEELNSTIKNVEQSAAISHQVSSKVKEEAD-RGRRVVKETIASLAEIQRSVELSFDAMKRLTENSGRIESIVGVINDITKRTNLLALNASIIAAQAGEYGKSFGVVADEIRNLSLQTGQSTGEITGIIEEIMNESHSAAQNISASKELVQKGVELGGVMGQSLQVIHESSARSLDMTQEIKIATEEQVRSVQLVTHSIENVSSMSSQIFKASKEQSDAAMS--------------IVRSVDTIKEMTQEMVKATVKQVEDGSEIKQSVEAVGEMVTKIFEDMEVRRGESSAVVRELEMMKKIAE-----------------------------------------------------------------------------------------------------------------------
```

```
Tar_E.coli     -LAQSVSHMQRSLTD--------------TVTHVREGSDAIYAGTREIAAGNTDLSSRTEQQASALEETAASMEQLTATVKQNADNARQASQLAQSASDTAQ-HGGKVVDGVVKTMHEIADSSK--------------KIADIISVIDGIAFQTNILALNAAVEAARAGEQGRGFAVVAGEVRNLASRSAQAAKEIKALIEDS--------------VSRVDTGSVLVESAGETMNNIVNAVTRVTDIMGEIASASDEQSRGIDQVALAVSEMDRVTQQNASLVQESAAAAAA--------------LEEQASRLTQAVSAFRLAASPLT------------NKPQTPSRPASEQPPAQPRLRIAE-------------------------QDPNWETF------------------------------------------------------------------------------------------------
```

```
Tsr_E.coli     -LAESLRHMQGELMR--------------TVGDVRNGANAIYSGASEIATGNNDLSSRTEQQAASLEETAASMEQLTATVKQNAENARQASHLALSASETAQ-RGGKVVDNVVQTMRDISTSSQ--------------KIADIISVIDGIAFQTNILALNAAVEAARAGEQGRGFAVVAGEVRNLAQRSAQAAREIKSLIEDS--------------VGKVDVGSTLVESAGETMAEIVSAVTRVTDIMGEIASASDEQSRGIDQVGLAVAEMDRVTQQNAALVEESAAAAAA--------------LEEQASRLTEAVAVFRIQQQ----------------QRETSAVVKTVTPAAPRKMAVAD-------------------------SEENWETF------------------------------------------------------------------------------------------------
```

```
Gmet1078       -LLRSLREMNDSLSH--------------IVAEVRTGADSIASATEQISAGNADLSQRTEEQASALEETASSMEELTSTVKQNADNAQQANKLAVTASEVAE-RGGEVIGRVVNTMGAITDSSR--------------KISDIIGVIDGIAFQTNILALNAAVEAARAGEQGRGFAVVAGEVRNLAQRSAAAAKEIKSLIEDS--------------VAKVEDGSRLVEEAGETTREIVTSIKRVADIMAEISAASIEQSSGIEQVNTAITQMDDVTQQNAALVEEAAAAAEA--------------LEDQARSMVSTVSRFKLADGGRADTGAAPKTERKPAARVAPSARNTPAAKAAPKAKAANG-----YHKPAAPEHHEAELPKAVGYDDDWKEF------------------------------------------------------------------------------------------------
```

```
Gura2167       -LLEALKEMNGSLAH--------------IVGEVRTGADSIATATEQISAGNTDLSQRTEEQASALEETASSMEELTSTVKQNADNAQQANQLAVTASDVAV-KGGDVIGKVVTTMESISDSSR--------------KIADIIGVIDGIAFQTNILALNAAVEAARAGEQGRGFAVVASEVRNLAQRSAAAAKEIKALIEDS--------------VGKVEAGSRLVEDAGRTTQEIVTSIKRVADIMAEISAASMEQSSGIEQVNTAITQMDDVTQQNAALVEEAAAAAES--------------LKEQARSMVDAVSRFKLNDSEQSPLKRVEVKLNNQTSAQAPANKSINQIKRPEKVRVANG-----YGKAAGPG--EVKLPKVVGLEEDWKEF------------------------------------------------------------------------------------------------
```

```
GSU0683        -VYAAMHNMVEKLKG--------------VVADVKSAADNVAAGSQELSSSSEEMSQGATEQAAAAEEASSSMEQMSSNIRQNADNATQTEKIALKSASDAK-QGGTAVAETVVAMKEIAS---------------------KISIIEEIARQTNLLALNAAIEAARAGEHGKGFAVVAAEVRKLAERSQKAAGEISELSAS---------------------SVQVAEDAGEMLTRIVPDIQRTAELVQEISAACKEQDTGAEQINKAIQQLDQVIQQNASASEEMASTSEE--------------LASQAEQLQATISFFRTDDRG--ASSRSAARRPVAKKKAAISHLGHGMSNGYHTEP-------ATSRKVAVGGGVDLNLDT-DHLDDQFEKF------------------------------------------------------------------------------------------------
```

```
GSU1298        -LLAAMHNMVEKLKG--------------VVADVKSAADNVAAGSQELSSSSEEMSQGATEQAAAAEEASSSMEQMSSNIRQNADNATQTEKIALKSAADAK-QGGTAVAETVVAMKEIAS---------------------KISIIEEIARQTNLLALNAAIEAARAGEHGKGFAVVAAEVRKLAERSQKAAGEISELSAS---------------------SVQVAEEAGEMLTRIVPDIQRTAELVQEISAACKEQDTGAEQINKAIQQLDQVIQQNASASEEMASTSEE--------------LASQAEQLQETIAFFKTGEQVGLVRKAAAVRQFAAKKKAAIPHLGHGTSNGYHAEP-------ATSRKVAVGGGVDLNLDS-DHLDDQFEKF------------------------------------------------------------------------------------------------
```

```
GSU1300        -LLAAMHNMVEKLKG--------------VVADVKSAADNVAAGSQELSSSSEEMSQGATEQAAAAEEASSSMEQMSSNIRQNADNATQTEKIALKSATDAR-EGGKAVAGTVSAMKEIAS---------------------KISIIEEIARQTNLLALNAAIEAARAGEHGKGFAVVAAEVRKLAERSQKAAGEISELSAS---------------------SVQVAEEAGEMLTRIVPDIQRTAELVQEISAACKEQDTGAEQINKAIQQLDQVIQQNASASEEMASTSEE--------------LASQAEQLQATISFFRTDDRG--ASSRSAVHRPVAKKKAAIPHLGHGTSNGYHAEP-------ATSRKVAVGGGVNLNLDS-DHLDDQFEKF------------------------------------------------------------------------------------------------
```

```
Gmet2424       -LLEAMANMVEKLKI--------------VVADVKSASDNVAAGSQELSSSSEEMSQGATEQAAAAEEASSSMEQMSSNIRQNADNAQQTEKIALKSATDAK-EGGKAVGQTVNAMKEIAG---------------------KISIIEEIARQTNLLALNAAIEAARAGEHGKGFAVVAAEVRKLAERSQKAAGEISELSAT---------------------SVDVAEKAGEMLERLVPDIQRTAELVQEISAACKEQDTGAEQINKAIQQLDQVIQQNASASEEMASTSEE--------------LASQAEQLQATIGFFKVDGSVS---GRSASVRKPPAHKVEVKHIASHSANGYVASA-------STRKAGSAGVDLDLSSES-DNLDKEFEKF------------------------------------------------------------------------------------------------
```

```
Gura2779       -LLAAMGNMVEKLKD--------------VVVNVQSAADNVASGSQELSSGSEEMSQGSSEQAAAAEEASSSMEQMSSNIRQNADNALQTEKIAVKSASDAR-AGGKAVEQTVHAMKDIAG---------------------KIGIIEEIARQTNLLALNAAIEAARAGEHGKGFAVVASEVRKLAERSQKAAAEISELSAS---------------------SVDVAERAGELLTKMVPDIQRTAELVQEISAASREQDTGAEQINKAIQQLDQVIQQNSSASEEMASTAEE--------------LASQAEQLQSAIAFFRVDEQ-GRGAMAGLPAQAAKPATKAVRSGKVTQITHLDHG--------AAMKKAVGAEGVAFNMGD-AAIDDQFEKF------------------------------------------------------------------------------------------------
```

```
Gura2985       -LLTAMGAMVAKLKE--------------IVGEVKSAADNVASGSQELSSGSEEMSQGASEQAAAAEEASSSMEQMSSNIRQNADNAIQTEKIAVKSAGDAK-EGGKAVEETVHAMKEIAG---------------------KISIIEEIARQTNLLALNAAIEAARAGEHGKGFAVVASEVRKLAERSQKAAAEISELSAS---------------------SVDVAEKAGDLLTKMVPDIQRTAELVQEISAASREQDTGAEQINKAIQQLDQVIQQNAGASEEMASTAEE--------------LASQAEQLQATIAFFKVDERSAVKRTAVQSRPVAKNAAKQTGKAKVHNIAHIKKANGYDKGQVANGKAAVNAGGVGLDMEN-DNLDSEFEKF------------------------------------------------------------------------------------------------
```

```
GSU1304        QLVAALNDMVAKLRD--------------IVTDVKNSADNVAAGSQELSSSSEVMSQGATEQAAAAEEASSSMEQMAANIRQNADNASQTEKIALKSATDAR-EGGKAVAGTVSAMKEIAS---------------------KISIIEEIARQTNLLALNAAIEAARAGEHGKGFAVVAAEVRKLAERSQKAAGEISELSAS---------------------SVQVAEEAGEMLARMVPDIQRTAELVQEISAACKEQDSGAEQINKAIQQLDQVIQQNASASEEMASTSEE--------------LAGQAEHLQSTITFFKTDEQGRAAGRSPAVRPAAVAKKPAALRLGHGNERRTEPVAP---------RKAVAGKGVDLKMDG-DYLDDQFEKF------------------------------------------------------------------------------------------------
```

```
GSU1294        ELMQALSAMVKKLSE--------------VVAEVKSAANNVAAGSREMSSGSEQMSQGATEQAAAAEEASSSMEEMSSNIRQNADNASQTERIAIKSAQDAR-DGGKAVAETVTAMKDIAS---------------------KISIIEEIARQTNLLALNAAIEAARAGEHGKGFAVVAAEVRKLAERSQKAAGEISDLSAS---------------------SVEVAEKAGEMLGRIVPDIQKTAELVQEISAASKEQDTGAEQINRAIQQLDQVIQQNASAAEEMASTAEE--------------LSAQSEQLQSIISFFRVDS---SAQSSSAIAAAKPAAKKPALAHAPANGYHKANQAP---------AKKVAHAGLNLNLEGGDHLDSEFETF------------------------------------------------------------------------------------------------
```

```
Gmet2423       ELMRALASMVARLRD--------------VVRDIVSAADNVGSGSQQLSSTSEEMSQGATEQAAAAEEASSSMEQMAANIRQNADNATQTERIATKSAADAI-EGGKAVGNTVQAMKDIAG---------------------KISIIEEIARQTNLLALNAAIEAARAGEHGKGFAVVASEVRKLAERSQRAAGEISELSSS---------------------SVEVAVRAGELLATIVPDIQRTAELVQEISAACREQDTGAEQINKAIQQLDTVIQQNASASEEMSSTSEE--------------LASQAEQLQTTIGFFRIGDEERRRPEARLTRAAKRIHVGHMDAGNAALPAKNRGDQP-----------PLAVGGISYDMGSGDSMDTEFEKF------------------------------------------------------------------------------------------------
```

```
GSU1140        ELMKALASMVTKLRD--------------VVADIMIAADNVTSGSQQLSSTSEEMSQGATEQAASAEEASSSMEQMSSNIRQNADNAAQTERIAIKSAADAI-EGGKAVGNTVSAMKEIAS---------------------KISIIEEIARQTNLLALNAAIEAARAGEHGKGFAVVASEVRKLAERSQKAAGEISELSSS---------------------SVEVAVRAGELLATIVPDIQRTSELVQEISAACREQDTGAEQINKAIQQLDQVIQQNASAAEEMSSTAEE--------------LSSQAEQLQDTVAFFSIGGEMKRKIAPKPSRPNAKASIRLPAAPHGTANG--YGRTS-----------ASVTGGFALDMAGHDHLDNEFEKF------------------------------------------------------------------------------------------------
```

```
Gura2992       ELMQALAIMVKKLTE--------------IVSEVKSAADNVASGSQELSSSSEEMSQGASEQAASAEEASASMEEMTSNIRQNADNAMQTEKIAVKSASDAK-EGGEAVTQTVIAMKEIAG---------------------KISIIEEIARQTNLLALNAAIEAARAGEHGKGFAVVASEVRKLAERSQKAAAEISDLSAT---------------------SVDVAEKAGQLLTKLVPDIQKTAELVQEISAGSREQDTGAEQINKAIQQLDQVIQQNAGASEEMASTAEE--------------LASQAEQLQCSIAFFKIGEESVGRKTAAVKKDDRKPAVKSRAK--DTMKHAMANGYA-----------KKAVGHDLEMGEDNEQLDNDYEKF------------------------------------------------------------------------------------------------
```

```
Gmet2422       ELMQALSEMVRQLTA--------------VVTEVKTAADNVASGSAQMSSGSEEMSQGATEQAAAAEEASSSMEEMSSNIRQNADNAAQTEKIAMKSAADAK-AGGEAVAETVVAMKDIAG---------------------KISIIEEIARQTNLLALNAAIEAARAGEHGKGFAVVAAEVRKLAERSQKAAGEISQLSSA---------------------SVEVAERAGEMLGRMVPDIQRTAELVQEISAASREQDTGAEQINKAIQQLDQVIQQNAGAAEEMASTAEE--------------LSAQAEQLQSTISFFRLKNEGSRNPASKLAKTRNRVQVGHIAADNGHSGKLAPGTAK-------------GYAFEMEN---SDAMDAEFEKF------------------------------------------------------------------------------------------------
```

```
Gura2989       ELMLALHAMVKKLND--------------VVSEVKSAADNVAAGSQELSSSSEQMSQGASEQAAAAEEASSSMEQMSSNIKQTADNALQTEKIAVKSAIDAQ-EGGKAVVQTVAAMKEIAG---------------------KISIIEEIARQTNLLALNAAIEAARAGEHGKGFAVVASEVRKLAERSQKAAAEISELSST---------------------SVEIAEKAGEMLTRMVPDIQKTAELVQEISAGSKEQDAGAEQINKAIQQLDQVIQQNAGASEEMASTAEE--------------LASQAEQLQCSIAFFKIGEESVGRKTAVVKKDDRKPAVKSKVKDTMKHAMANGYAKK-------------AVGHDLDMNEDSEQLDNEYEKF------------------------------------------------------------------------------------------------
```

```
GSU1303        -LLAAMGNMVSKLRE--------------VVTSVKSASDNVAAGARELSVSAEEMSEGATEQAAAAEQASGNMEEMSGSIRHTADNAVQTEKIAGKSAADAR-EGGEAVAETVSAMKVIAG---------------------KIAIIEEIARQTNLLALNAAIEAARAGEHGKGFAVVASEVRKLAERSQKAAGEIGELSAS---------------------SVRIAEKAGEMLARMIPDIQRTAELVQEISAACKEQDSGADQINRAIQQLDNVIQQNASTSEEMASTSEE--------------LASQAEQLQATIAFFNI---------------------------------------------------------------------------------------------------------------------------------------------------------------------------
```

```
Gura1191       EMMKDLATMVKKLSD--------------VVQDVMSAADNVAAGSKELSANSEHTSQGASEQAAAAEEASSSMEQMSSNIKQTAENAMQTERIAVKSAEDAQ-EGGKAVASTVTAMKEIAG---------------------KINIIEEIARQTNMLALNAAIEAARAGDHGKGFAVVAAEVRKLAERSQKAAGEISELSVS---------------------SVEIAEKAGELLGAILPNIQKTAELVQEISAASREQDSGADQINKAIQSLDQVIQKNAAVAEEMASTAEE--------------LSSQAGQLQGTISFFRVDETANKKKNTILKPAPATDLKPSAHTVRKEYRQPLKKAVG-------------------AEGITLNLEDEEFERY------------------------------------------------------------------------------------------------
```

```
GSU1141        -LAVGINAMVGRFRD--------------VVTSICRDSEAVAGAASQLSGTACQLSEAATEQAAAAEDASSSMEQISSAIRANVQNAQTTADVANRSSIDAA-AGGETVTETVALMKEISR---------------------KIMVIEEIARQTNLLALNAAIEAARAGDHGKGFAVVAGEVRKLAERSQSAAAEIGRLSVT---------------------SVEVAERAGTLFGAIIPDIRQTAELVQGISSACHEQETGVGQINRAIRQLDAVIQQNASASEQMASTAQE--------------LSSQADMLLDAVSFFRLGETNRYQESRSELSGIS----------------------------------------------------------------------------------------------------------------------------------------------------------
```

```
GSU1029        -LAREVNTTAAKINE--------------IIGLVAHNASQVTAAATQLHATSTQMSTGAEEVAQQAATVATASEEMAATSAEIAHNCSLAAESSRHANDRAE-NGSDVVQETLTVMNRIAERVKDSARTVESLGERSDQIGEIIGTIQDIADQTNLLALNAAIEAARAGEQGRGFAVVADEVRALAERTTKATKEISQMIKAIQGETKGAVTSMEEGVKEVEKGTSDASKSGEALQAILEQIGGVTMQVSQIATAAEEQTATTGEINNNIQQITEVVQLTARGAEESAQAAEQ--------------LAKLAEELQDLVYKFKLA--------------------------------------------------------------------------------------------------------------------------------------------------------------------------
```

```
Gmet0712       -LSRQVNTTAEKMNE--------------IIGHVTQNASQVTAAATQLHATAIQMSTGAEEVAQQAATVATASEEMAATSAEIAQNCTMAAESSRHANARAE-TGSSVVQETLTVMNRIADRVRSSSQAVGSLGARSDQIGEIVGTIEDIADQTNLLALNAAIEAARAGEQGRGFAVVADEVRALAERTTKATKEIAQMIKAIQGETKGAVTSMEEGVKEVEKGTTDASLSGEALQAILEQIGGVTMQVSQIATASEQQTATTCEISENIRQITDVVGHTARGAEESAQAAEQ--------------LARLAEDLQVLVGQFTLAA-------------------------------------------------------------------------------------------------------------------------------------------------------------------------
```

```
GSU0756        -LGRELNVTAEKIGK--------------IIGQLAQAAGSVASASAQLHATAEQMATASEEVAAQAETIATAGEEMAATSNDIAHNCVTAAEGSTQANDAAE-GGAQVVQAAIAAMDRIAERVHASAKTVEGLGVRSEEIGEIIGTIEDIADQTNLLALNAAIEAARAGEQGRGFAVVADEVRALAERTSKATRQISEMIRAIQHDTQSAVHSMEEGVSDVQAGTAEAARSGQALQMILAKIGDVTNQISQIATAAEEQTATTGEISNNMHQISQVVQDTARGAQDTVAAANS--------------LSRLSEDMQGMVQQFRLA--------------------------------------------------------------------------------------------------------------------------------------------------------------------------
```

```
Gura2845       -LASWFNIFIDKLHS--------------IISQVTNNTIRVASAAGQLSSTSEQMAAGFEEVAAQAGTVATAGEEMAATSTEIAQNCNMAAQGSQQANKAAM-NGAKVVAGTVQVMNRIAGRVRDTAKTIEGLGDRSDQIGEIIGTIEDIADQTNLLALNAAIEAARAGEQGRGFAVVADEVRALAERTTKATREIGEMINAIQNETKGAVGIMEESVKEVENGTFEAAKSGQALQDILDQINSVAMQVNQIATAAEEQTATTAEISNNIQQITGVVHETAKGAQESAHAASQ--------------LSHLSEELQSLVGHFKLVA-------------------------------------------------------------------------------------------------------------------------------------------------------------------------
```

```
Gmet2828       -IGGSFNETADSFAR--------------VIAGIRGNAEQVATAATQVHSSAEQMATGVEEVAAQTGTVATAGEEMAATAAEIAQNCQMAAEAAQRATESAT-GGATVVQRTVNGMARIADRVRSSAKTVESLGSRSEQIGEIIGTIQDIADQTNLLALNAAIEAARAGEQGRGFAVVADEVRALAERTTKATREIGEMIKAIQQETRGAVAAMEEGVHEVEAGTADAQQSGAALQEIMNQINELAMQVSQIATAAEQQTATTGEISGNVQQVSEVVQETAKGIQESAQAASR--------------VAELADELNNLVGRFKVA--------------------------------------------------------------------------------------------------------------------------------------------------------------------------
```

```
GSU2942        -LAEAVNTMADRLNR--------------LIAGVAENASQVAAAASQLTSNAEQMATGAEEVAAQTGTVATASEEMASTSAEIAQNCTAAAEESRRASDTAV-QGSEVIRHTVGEMERIAERVRETARTVESLGARSDQIGEIIGTIEDIADQTNLLALNAAIEAARAGEQGRGFAVVADEVRALAERTSRATREISTMIKAIQQETKGAVASMEQGVREVERGTAEASQSGKALEEILEQVGCVTMQINQIATAAEQQTSTTSEISGNIQQITDVVQQTARGAQETAAAARQ--------------LSQLSAELQHLIGQFHLAA-------------------------------------------------------------------------------------------------------------------------------------------------------------------------
```

```
Gmet0529       -LAEAVNTMSDRLNS--------------LITGVADNAAQVAAAAGQLTANAEQMATGAEEVAAQTGTVATASEEMAATSTEIAASCSAAADEARRASETAG-KGSEVIKQTVGEMHHIAERVKETAKTVENLGTRSDQIGEIIGTIEDIADQTNLLALNAAIEAARAGEQGRGFAVVADEVRALAERTTKATKEIGAMIKAIQQETRDAVSFMEQGVKEVELGTAEAAQSGKALVEILDTVEAVTMQVNQIATAAEQQTATTTEISGNIQQITDVVQGTARGAQETAAAARQ--------------LSDLSSELQHLIGQFHLAS-------------------------------------------------------------------------------------------------------------------------------------------------------------------------
```

```
GSU1374        -LMAAMGNMVTSLRH--------------LIAEAISISHGIASASNQLHATSEQIATGSEEVASQVGAVATASEEMSSTSRDIAQNCTLAAESSRETSVTAS-NGSAVVQETNSGMVVIAERVKQTAGTVDALGRRSEQIGEIIGTIEDIADQTNLLALNAAIEAARAGEQGRGFAVVADEVRALAERTTKATKEISGMIKAIQNETKAAVQAMEEGVGEVEKGSVTSHKSGQALAEILDRINDVTMQINQIATAAEEQTATTGEITSNIQQISDVVQQTARGAEEVSAAAAQ--------------LAQQAHQLQNVVGNFRIA--------------------------------------------------------------------------------------------------------------------------------------------------------------------------
```

```
GSU2579        -LLTAMQNMVRSLRE--------------MVTQTATISAGIASASSQLHATSEQIATGTEEVASQAGTVATASEEMSATSQDIATNCHAAAGSAEQVAATTR-QGFDVVRHTVDGIRDRGEKTRQNAQIVASLGDRSEQIGDIVGTIEDIADQTNLLALNAAIEAARAGEQGRGFAVVADEVRALAERTTRATKEIGEMIRAIQQETKTAIVSMEEGVRGTERGAIEAAQLETALQQILNQVNEVSMQVGQIATAAEEQTATTGEVTSNIQQITEVVHQTAQGAEETADAAAQ--------------LARQAQDLQALIGRFRLA--------------------------------------------------------------------------------------------------------------------------------------------------------------------------
```

```
GSU1041        -LAVSVNRMADDMGT--------------AMAALANASSHLASASVELAVQADQMAKGAEEVAAQTGTVAAASEEMAATSHEIAMNCSHAAESSRRANDRAS-AGSDVIRRTVEGMHRIAEKVQRSSESVAGLGARSDQIGQIVSVIEDIADQTNLLALNAAIEAARAGEQGRGFAVVADEVRALAERTGKATREIAQMIRSIQQETEGAVKAMEEGVAEVSAGKEDAQQSAGALREIVEQIEAMTTQINQIAVASEQQNATTDQITMNLQQVSSVIEASSRGSEETANAAHT--------------LSALSEELQSIVGRFRTAA-------------------------------------------------------------------------------------------------------------------------------------------------------------------------
```

```
Gura0612       -LGRSINRMLTSIAG--------------MITSIKNTATQVASAAGVLYSNSEQIATGAEEVAAQAGTVATASEEMAATSSEIAQNCNLAADSSRHASRLAT-EGVSVVHETVAGMNRIAERVKESAATVESLGSRSDQIGEIVGTIEDIADQTNLLALNAAIEAARAGEQGRGFAVVADEVRALAERTTKATKEIGQMIKAIQTETKGAVTSMEEGVNEVELGTKDAAKSGSALEHILNQINEVTMQINQIATAAEQQTATTTEITHNIQQITEVVQASTSGSHASAYSASE--------------LTAHAEELQRLVGQFTLAA-------------------------------------------------------------------------------------------------------------------------------------------------------------------------
```

```
GSU0750        -LCTEFNSFVGKVHD--------------TISRTSSVARDVTGSVAEISRTAERLAEGAEEVASQAVMAATASEEMAATSCEIAGNCQTAAQSSSRARETAA-RGFAMVENTIAVMNQIARRVRVSAESVQGLGARSDQIGEIVMTIQDIADQTNLLALNAAIEAARAGEQGRGFAVVADEVRALAERTSRATREIGEMIKGIQGETRTAVLTMEEGVKEVEAGTREAAKSGEALNEIMQGIEQLNQQMGQIACAAEQQTSTTMEISGSIQRIKDVAQETAGGAHDSARTSTR--------------LTDLSHDLDRLVSQFRV---------------------------------------------------------------------------------------------------------------------------------------------------------------------------
```

```
GSU1033a       -ISAWFNTFIDKLHG--------------IISRVAQTTAEVASAAAHVYDTAEQMATGAEEVAAQSGTVATASEEMAATSAEIAQNCSLASEGANRASQSAD-DGAGVVEATVRVMGYIAERVNVAARTVEKLGERGVQIGEIIETIEDIADQTNLLALNAAIEAARAGEAGRGFAVVADEVRALAERTTRATREIAGMIKAIQSETNDAVSSMEEGVRDVEAGTGEAARSGEALKDILSRINDVVHEVHQIATAAEQQTATTNEISRNMQEITDVVQQTAHGAQESALAAER--------------LKRQAEELQRLVGQFRLAT-------------------------------------------------------------------------------------------------------------------------------------------------------------------------
```

```
Gmet0821       -LGRSFNLFVEKLQK--------------TIAMVADNTAQVAAAAGQVYSSSEQMATGAEEVAAQAGTVATASEEMAATSNEIANNCMLAAEGSREASGSAS-GGSQVVEQTVAVMNRIAERVKEAAHTVEGLGARGDQIGEIIGTIQDIADQTNLLALNAAIEAARAGEQGRGFAVVADEVRALAERTTKATREIGEMIKGIQQETGRAVSSMEEGVKEVASGTAEAAKSGDALREILDRITGVTGQVSQIATAAEEQTATTAEITNNIQQITTVVEATARGAQESATAASR--------------LTDLATELQGLVGQFKV---------------------------------------------------------------------------------------------------------------------------------------------------------------------------
```

```
Gura3311       -MGGAFNTFIEKLHG--------------IISQVAQSTVQVAAAASQLYSSSVQMATGAEEVAAQSGTVATASEEMAATSTEIAQNCTYAAEGAKQANGSAM-TGAAVVETTVDVMGRIAERVRESAQTVGSLGERSDQIGAIIGTIEDIADQTNLLALNAAIEAARAGEQGRGFAVVADEVRALAERTTKATREIGTMIKAIQGETKGAVTAMEDGVREVEKGTAEAARSGDAIQDILNQINAVSMQVNQIATAAEEQTATTCEINNNMQQITEVVYETAKGAQESATAASQ--------------LASLAEGLHRLVGQFKLA--------------------------------------------------------------------------------------------------------------------------------------------------------------------------
```

```
GSU0401        -LAVSFNNFVGKLHD--------------IIAQVSQGTLQVASASYELQANAEQMAHGAEAAATQVNTVASSSEVLAASTFEISSNCGTVAESSRRANDSAQ-TGAVVVEKTVDIMARIAERVKDSARTVESLGARGNQIGEIISTIEDIADQTNLLALNAAIEAARAGEQGRGFAVVADEVRALAERTSRATREISQMIKGIQGETRGAVLAMEQGVKEVELGSEEAARSGEAIRTILEQFRTLDCQVGEISAAAEDQTRVTTEISTNVMQITEIIETTAKGAADSAEAAQG--------------LAELSDQLKQIVGRFKLSV-------------------------------------------------------------------------------------------------------------------------------------------------------------------------
```

```
GSU1035        -LAGDMNRMVEKLRD--------------MVAGVAGAAAEVTTAARQLSSTSEEMAAGVQSAAAEVVGVSTAGEEMAATSFEISFNCSTVAADARQATESAT-AGEEVVSATVCIMANIAALVRDSARTVESLGARSDQIGELAGSIEDIADQTNLLALNAAIEAARAGEQGRGFAVVADEVRALAERTARATREITAVIRSIQQETQGAVTAMTAGVVEVERGTAEASRSGEALRGILERIHAVEEQVVQIAAAADQQTATTTEISGNILRISDVVQSTTRGAQDSADAAAH--------------LQGLAEELHAAVGRFRVAG-------------------------------------------------------------------------------------------------------------------------------------------------------------------------
```

```
GSU1033        -MSRSFNSFMDKLHG--------------IITHVARTVEQLASSASQVHGSAEQMAAGAGEVASQAGTVATAGEEMAATSTEIAQNCAMAAEGARRASSTAT-AGAEVVGNTVTVMDRIAEKVKNSARTVERLGERSDQIGEIVGTIEDIADQTNLLALNAAIEAARAGEAGRGFAVVADEVRALAERTTKATREISGMIRAIQAETLEAVSSMDEGVRDVETGTAEAARSGEALREILDQITAVSMQVNQIAVAAEQQTSTTREISGNIQQITEVVEGTAQGADESACAAGG--------------LNRLAEDLQRMVGQFRL---------------------------------------------------------------------------------------------------------------------------------------------------------------------------
```

```
Gura3063       -MCTSFNLFMDKLYT--------------IVSRVAGTTSQLAAAALQVQGSSNQIAEGADKVAQQSATVATASEEMALTSSEIAQNCLLAADEARQATDSAQ-TGSVVVEQTINKMNSVSEQVMAAARTVESLGQRSDEIGEIIGVIEDIADQTNLLALNAAVEAARAGDQGRGFAVVADEVRALAERTTKATRRIADMIATIQKETNAAVISIKQGVEEVEAGASEAHRSGDALRDILRQIDALNRQFNQIATAAEEQTSTTSEISRSMHQITDIVHDTAKGTQDAAAAAMQ--------------LNQLAQTLQSLVGQFRL---------------------------------------------------------------------------------------------------------------------------------------------------------------------------
```

```
GSU1032        -MGRTFNRMVENFEH--------------MLTSIQNAVLNLSESARTLSVTSEQIATGAEEMASQTGTVATASEEMAATSQEIAQNCSTAADVARNASASAR-SGAAVVQQTIGAMERITERVRDTARTVEALGARSDQIGEIVGTIQDIADQTNLLALNAAIEAARAGEQGRGFAVVADEVRALAERTTRATREIAEMIKSIQQETRGAVASMEEGVVEVTQGSADAARSGDALREILDQIEQVTGQVAQIATAAEQQTATTSEITMNIQQITEVVGHTAREAGESADAATG--------------LATLADELQTEVRTFKTSGSELFILELAKKDHSGFVTTVEAVLVGRRRMEAGELSTHHTCRFGKWYEGDGRQLCGHLASYKAIYAPHERIHSLARDVVAAVNGGDRDRAARLFPELKELSREIITRLDDIRREFEAQRAAA-----------------------------------------------
```

```
GSU1030        -LGRAFNQFIEKLHN--------------IISQVVQNSMQVASAAAQIHSTSEQTATGAEEVAAQAGTVATAGEEMASTSSEIARNCMAAAENSRQANDTAL-KGSHVVKETLTVMTRIADRVKESAHTVESLGSRSDQIGEIVGTIQDIADQTNLLALNAAIEAARAGEQGRGFAVVADEVRALAERTTKATKEIGQMIRSIQQETKLAVSSMEEGVKEVERGTSEAAKSGEALEEILHQIGEVTNQVNQIATAAEQQTATTSEISSNIHEITEVITQTTRGAQDSASATSD--------------LARLAEELQRLVGQFRLS--------------------------------------------------------------------------------------------------------------------------------------------------------------------------
```

```
GSU0400        -LALAFNRFVEKLQG--------------IVGTVANNALQVAAAAGQVQEASRQMAEAAENVAGQAATVATASEEMAATSMEIAGNCVSLADGARHASETAE-SGAAVVQETVSVMGRIAERVKEAARTVDSLGSRSDQIGEIIGTIEDIADQTNLLALNAAIEAARAGESGRGFAVVADEVRALAERTTRATREIALMIKAIQNETRGAVASMDEGVREVEKGTGEAARSGAALREILEQIGSVSLQISQIATAAEQQTSTTTEISGSIQTITDTAHETARGAQESAGAAGQ--------------LADLAEQLQNVVMTFRLSA-------------------------------------------------------------------------------------------------------------------------------------------------------------------------
```

```
Gmet2825       -LARSFNRFLDNMKE--------------IVQRINQNAVDVASSADHLNETAGHIASGTERASTQSTSVAISCEEMAATSSEIAHNCIRTVEIANRATQTAQ-DGSLVVSHAVSSIQRIAHKVQESAKTVESLGVRSEQIGNIVGTIEDIADQTNLLALNAAIEAARAGEQGRGFAVVADEVRALAERTTKATREICEMIKSIQQETKFAVAAMEEGVQEVEKGTAEAGRSGEALEEILAQVAELTSQINQIATAAEQQSATTNEISKSMYEITAVISDASGSSQNTANAASQ--------------LAGMADELKRIVAQFRM---------------------------------------------------------------------------------------------------------------------------------------------------------------------------
```

```
GSU2652        -IIRSIGELQSTMRE--------------IISRISQTSQEVALASRQLQANADQIASGTENVASQANTVAVASEEMAATSSDIADNCLSAADNSTRASTTAR-SGSEVVRRTTDCMERIADKVKGAARTVEGLGSRSDQIGQIIETIQDIADQTNLLALNAAIEAARAGEQGRGFAVVADEVRALAERTTRATREISQMIKSVQTETKEAISAIDEGVAEVEKGTEYSGESARSLDQILQQISDVTQQINQIATAAEQQTSTTAEISNNIQQITAVVDQTAQGATETAGAAAT--------------LSRQSEELQRLVGQFKL---------------------------------------------------------------------------------------------------------------------------------------------------------------------------
```

```
Gmet0799       -IIRSIASLQATIRE--------------IVSQISQTSEEVAMASRQLQSNADQIAAGTDNAASQTNTVAVASEEMAATSGDIANNCMRAAENSTRAVNTAR-SGAEVVRQATDCMERIASRVRDAAKTVEELGSRSDQIGQIIGTIQDIADQTNLLALNAAIEAARAGEQGRGFAVVADEVRALAERTTRATREIGEMIKTIQNETKGAVSAIDEGVAEVERGAEYSDKSGQSLEQILQQINDVTMQINQIATAAEQQTATTGEISSNIQQITAVVQQTARGATETATAAAT--------------LSRQADELQGLVGHFRL---------------------------------------------------------------------------------------------------------------------------------------------------------------------------
```

```
GSU0582        -LAEGINLLVTKLRE--------------IISGLYHQAGHIAISACRTIKETERLVASTHEQKDLSTSVAVASEEMAATLNDVAVNTQRAAQLSLSVDRAAH-EGMATVTETAESIDRIKDSVMATLDTMDKLQQSSGQIGEIVGIIGDIADQTNLLALNAAIEAARAGDSGKGFAVVANEVKVLSDRTASSTREIGTIIRSIQAEIRAVVASIAEGKDKVEVGVERSTTARRQLEDILRLAAESTDMINQIATATEEQSATTGEISEKISQVSGTAERVNGQMEQTAGIFRE--------------LSETAEQIYGTVGRFKVGTYHDTVKGLASEMRDRVVATLERAASDRRVTLDALFSSEYTPIPDTFPQKYRTPSDRLFDEIISPIQEEILGRDSGMYYAICVDRRGYCPSHNLRYSRPLTGNREADKEHNRTKRIFEDRTGLRCAGNTGSFLLQTYLRDTGEVMNDLSVPIVIGGRHWGAVRIGYRADD
```

```
Gmet2939       -LAEGVNMLIAKLRE--------------IVTSLYSQAGHIAISACRTVKGTEGLVASTAEQKDLSTSVAVASEEMSATLNDVAATTQRAAQLSINVDQAAK-VGMETVEETSLSIDQIRTSVLGTLGAMGKLETSSGQIGEIVGIIEDIADQTNLLALNAAIEAARAGDAGRGFAVVANEVKTLSNRTATSTRQIAAIVRSIQEEIGTVVTSIGEGKTRVEEGVEKAGHARQQLEGILRLATDSTDMISQIATATEEQSATTVEITEKIGQVSATAGAVNGQMEETARIFRE--------------LSETAEKIYGTVGRFSVGCYHDAVKGYAAELRDRATAVIEKALEDRKITIDALFSTDYTPIPNTTPQKYRTPFDRFFDELVSPVQEEILGRDSGVYYAICVDREGYCPSHNLRYSRPLTGDVAVDKDHNRTKRIFNDRTGIRCATHTQSFLLQTYLRDTGEVMNDLSTPITVGGKHWGAVRIGYRADD
```

```
Gura4401       -LAVEINHLISKLRE--------------IISSLYQQAGNISVSVCTVALGANKTVSATTDQKEQAMSVAVATEEMAATLNVVASNTHRAAEFSAQVDSAAS-EGMTVVDEACNCIKIVNDNVATTLGTVERLETSSNKIGEIVVLIEDIADQTNLLALNAAIEAARAGEHGRGFAVVADEVKNLSAKTATSTKEIAKIITDIQNESREAASSIIEEKKRVEEGVEKSLAARDCLEKILQIAGESADMINQIASATEEQSATVNEISSKIHHVSETSTTVHTQMQTSGKAFEE--------------LSEVAEQIFSTVGKFSVGNHHDTMKNYACELRDRAVAAIEKAISEKRIRMEDLFDRNYQAIPKTSPQKYSTSFDKFFDQFISPLQEEIAAKSGEIFFAICVDDHGYVPCHNLRYTKPLTGDLETDKVNNRTKRIFDDRTGIRAAKSSDTSLLQTYMRDTGEIMNDMSTPIYINNRHWGAIRIGYKAK-
```

```
GSU0935        -LAGGINRLTSTIQG--------------IITRIAQNAAQLASAASQLNVTSADMARSMEAVAGQATTVATASEQMASTSQEIAGSCSIAADGAMQATETAR-DGAEVVERTIAVMASIADRVKDTARTVESLGSRSDQIGEIIGTIEDIADQTNLLALNAAIEAARAGEQGRGFAVVADEVRALAERTTRATKEIGSMIKSIQQETRGAVTSMEEGVHEVTRGTDEASRSGESLQAILQRVSDVTGQVNQIATAAEEQNATTGEITRNIQDITDTVQSTARGAQDSAQAAGQ--------------LASLARELQELVGKFKIGA-------------------------------------------------------------------------------------------------------------------------------------------------------------------------
```

```
GSU0916        -MLLTMRELQGSMRD--------------IISGIQTTAADLSAASDLLRTTSSQIAEGTDHASQESASITTAVDEMASVSLAISHNCQKMAEEASGTGHATE-SGTETISRMTTIMEAVEQMVSGTMAAVNALGANSERIGDIITAIRDIADQTNLLALNAAIEAARAGEQGRGFAVVADEVRNLAERTTSSTREIQSIIGALQGDVKNVMGLMEQSSDSVRNGTRDMHLSRQAIGAIKEHIAPLIDHVSQVAIAAEEQSATTASITENIHRIALVIRDAAQGAQQTETAAAD--------------LAQSATELQQMVNRFKLSA-------------------------------------------------------------------------------------------------------------------------------------------------------------------------
```

```
Gmet3087       -VQASMAKMVDSIRQ--------------IVARIGAATESLASNSEEMSTTAAVLEEGAEQQALRVEQSAAAMVEMSQTTLDVARNTSEAAGTAEMMKTAAL-EGKEAMSLTVRELRHFAGTFEDTAGKVELLGDQSAQINEIVTLIEDIADQTNLLALNASIEAARAGEQGRGFAVVADSVRQLAERTATATADISQTVKAMQESVKQSVGSMHHERRSVGTILDRINDTMEATDRIVEYVERVTDMVRRIAVAAEQQSSTSGDVSRNMDEISVITRELRSSFSDIKHSSED--------------LSRLAGELNGMVGWFRV---------------------------------------------------------------------------------------------------------------------------------------------------------------------------
```

```
Gura0476       -LAVHFNQATAKLKE--------------ITSQIREAIGNLAYSSSSLTATAEELSAGARQQATQTDQSASAMIEMSQTIQDVARNAHETAAETKNSLTLAS-DGQKIVGETVRGMEEIAASVKETADTVKLLGENSTRIGSVVDVINEIADQTNLLALNAAIEAARAGEAGRGFAVVADEVRKLAEKTGESTREIAEMVAQIQASTQKSVRAMEKGTAKVEEGMLRATEANLALESIVGASDKGVAMVQTIATAAEEQSAVAAEVSTSMEHIATITRSAEMSTGEITRAAEE--------------LNRLAGDLNRMAGWFKM---------------------------------------------------------------------------------------------------------------------------------------------------------------------------
```

```
Gmet2709       -LLQTFGRMVENLRR--------------QAMDIQEGVNVLAASAGEILASTTQVASSAAETASALNETTATVEEVKQTTQLAAQKARNVADTAQRSLQVSQ-GGRKAVESSVEGMNRIREQMATIAESIVNLSEQSHAIGEIIATVNDLAEQSNLLAVNASIEAAKAGEHGKGFAVVAHEVKSLADQSKQATAQVRALLNDIQKGTNAAVMATEQGSKAVEAGEKQAAEAGEAIQLLTESIAESANASAQITATSQQQLVGMDQVALAMENISEASALSVTSTKQAESSAQN--------------LHELGQKLKRLVEQLKL---------------------------------------------------------------------------------------------------------------------------------------------------------------------------
```

```
Gura4170       -LAEAFNKMTTVIVKNLKGEIDKSS---RLIASIREAIIRLSSSANEMMAISAQQSSGATQQATAVQEVTTTSEEIAITAKQITDNAKSVESMAEETTQSCT-AGTSDVTNAIEGMTILKTQVQSIAESMLQLGDNSQKIGGIVEIIDEISDQTNLLALNAAIEAAGAGEAGKRFAIVAQEVKRLAERTVDATKQIKGLIEEIQKATNSTIMVTEEGTKGVDAASALVDKVQLSFSNIINMVEETARAAKEITLSTQQQTSACEQMAETMTEVRDVAQQVATSATETERAISD--------------IMEQTEKLRDLSDKEA----------------------------------------------------------------------------------------------------------------------------------------------------------------------------
```

```
McpA-B.sub     -LGKSFNNMASSLRSLIHAIQDSVDNVAASSEELTASAAQTSKATEHITLAIEQFSNGNEKQNENIETAAEHIYQMNDGLTNMAQASEVITDSSVQSTEIAS-EGGKLVHQTVGQMNVIDKSVKEAEQVVRGLETKSKDITNILRVINGIADQTNLLALNAAIEAARAGEYGRGFSVVAEEVRKLAVQSADSAKEIEGLIIEIVKEINTSLGMFQSVNQEVQTGLDITDKTEMSFKRISEMTNQIAGELQNMSATVQQLSASSEEVSGASEHIASISKESSAHIQDIAASAEEQLASMEEISSSAETLSSMAEELRDMTKRFKIE--------------------------------------------------------------------------------------------------------------------------------------------------------------------------
```

```
GSU3196        -LGDSINIMVGSLRELVGHIRTTAEKVAASARTLSDSTVEVNSSSEEVAQAVEQIARGAGTQAEMVERSSRIIHEMAISVELVSKRARESAKAAQETSRTAR-RGQKLANDSLERMTSFFGKVEESSAQFLSFNARLQQVGKIADFIAEIARQTNLLALNASIEAARAGEYGKGFAVVADEVRKLADGTGKSAAHITELIAAVREESRRVQQLIEESSRDIGEGKRNVDITAGAFQDILSNALETERRAGSIADLSHIQTDGAQKMVTAVDEIARVAEDNAAATEEVSAASEQQAIAMHEMTVAARDLADLAGVLMGVVERFILPRSEGGNKAP-----------------------------------------------------------------------------------------------------------------------------------------------------------------
```

```
Gura0724       -MAVSINRMVGNLRELVKHIRGTSENVSESSRTISSSALEINASSEEVAQAVEQISRGAETQAEMVTKSSKVIHEMAISVDLIARRAREASKAARETSLTAQ-RGGDLAKDSLTRMKSFFDSVELIGMQFMDLNTKLQQVGKIADFIGEIARQTNLLALNASIEAARAGEYGKGFAVVAEEVRKLADGAGKSAADIIELIAMVKEESRRVHETIADSSRNISAGKKNIDTTAESFREILNTVIETERKTNSIADLSQMQTAGAEKMVSMIDEIAKVAEDNAASTEEVSAATEEQSAAMQEMVHATAELTKLAEELLTIVEQFKVPGGAQLAL-------------------------------------------------------------------------------------------------------------------------------------------------------------------
```

```
GSU2423        QAAGGSAESIKTAKS--------------VMDQMLQAMGQIRSAADNTVGIVQQIDSIAKETDQLSSNATSKATLIRSSANGFSVVASEIRNLSKRCEDAVT-RLHDFRRRATLTPNGGSGD---------TDDALECEYLELIHELKSVASSSGLLGVNAAISAAHVEGAGNDFQVLTEEIRQLAKRSTDAARQTDTLIKTS--------------VEQARRGEDLSRKIDVHLTEAVTGATTICALTEDISQSSQEQASAIEQISRSVNHITTITRQNADSALKSSEVSHK--------------LGQQMTKLTSMVSKFRLDNAAC----------------------------------------------------------------------------------------------------------------------------------------------------------------------
```

```
Gmet3324       QVAQQAAESIGTAKT--------------VMDQMLHAMGEIRGSADNTVGIVQQIDAIAKETDKLSSNATSKAALIRSSASGFSVVASEVRNLSKRCEEAVS-RLHDFRRRVTFTPNAGTDS---------TTDQLVSEYLDLIHDLKSVASNSGLLGVNAAISAAHVEGAGNDFQVLTEEIRELAKRSTDAAKQTDTLIRTS--------------VEQARKGEDLSQAIDGHLTTAVTGASTISSLTDEISQSSQEQASAIEEISCSVTHINEVTRQNAACALSSSEVAQG--------------LGQQVTKLSKMVSKFRLARAAGQ---------------------------------------------------------------------------------------------------------------------------------------------------------------------
```

```
McpC-B.sub     LMVENMKEMVEQVRLSSGK-------VSDTSEQLTAVAAETNERSGQIAKAIEEVAAGASEQASEVETINEKSESLSTKIRQIAEEAGGIKERSKSSEDASY-KGLHALGQLLMKSNEANMETKKEETMLLDLENQTKNIEEVVTAISNISDQTNLLALNRSIEAARAGESGRGFAVVADEVRKLAEQSALSTKHISETVKLIQLETKEASHAMVEASRMNDEQNSAIHETGEVLNTITAEMQSLVQGIDHIYAEIQRMSEEQLAISEAIQSISAISQESAAAAEEVNASTDE--------------QLVTLDKVKHSTETLKHASQDVINTIRKFTL-------------------------------------------------------------------------------------------------------------------------------------------------------------
```

```
Gura2483       VSLTEVGAASEEIAG--------------KVETLNTNINSSYSTVAELGQSAKNVAALAAKASMTVGGVSGSVAQIKESVKKIENSVKESVELSNDTTKVISDKGIVSVYETKASMEKIDLIVGTLSKSIGNLGSRSKDITRILAVIKEVTDETKLLSLNASIIAAQAGEHGKSFAVVANEIKLLSDKTVGSTIEIEAIVRAIQQDIDVAVRGTGETSKIVHEGGKVVSKAGDALREILGSARKSTEMIKSIEDSAVEQSDGIENIIGAVNELQTLNYEVNRATEEEEKSISHLVKGISSIKDAMEMTGRAANEQASTLQSIQFNLQAANDRTAEIVAASTQQQHVNGGIIVSMDQIMDIGASTISGFQGVSASIAAISIEIESLRREMLIFRTESKSADECTASGAV---------------------------------------------------------------------------------------
```

```
Gura4222       -ITAEMSATTDTIAEN--------------IKDYSSSVLETSASIEEMALSIKETTSNIEALAVSTEQTSGSINQINTVTTDMRDNAQKTSECSENVRKKAQ-EGMRSMTATLKSMQEIEKSNAESFAAINRLAVHSARVGEFLNVIKDVVEQTNLLSLNASIIAAQAGERGKAFTVVAEEVRSLAHRTALSAKEIEDLVKNIQKETADVQRTVAQGKDRIKEGVKISALASNALEKIEESAAEASQMVQKIAAATVEQASSSRLITDEAEKNFHRVKQVTKAIQEQERGISH--------------IVKALEHMRSLSQLITNSTQEQARGNRLYLKSIMEDNDKVKELKDTAIQQIMMGDVLVNYVREAGSLIEANAGEARQMMDQIDKITTMTEDLCKELAPFTSRPAIQ----------------------------------------------------------------------------------
```

```
 
```

```
highlight: putative methylation site
```

```
highlight: putative pentapeptide
```
